# Pharmacological treatment options for metabolic dysfunction‐associated steatotic liver disease in patients with type 2 diabetes mellitus: A systematic review

**DOI:** 10.1111/eci.70003

**Published:** 2025-02-12

**Authors:** Laura A. M. Konings, Lorena Miguelañez‐Matute, Anna M. P. Boeren, Inge A. T. van de Luitgaarden, Femme Dirksmeier, Rob J. de Knegt, Maarten E. Tushuizen, Diederick E. Grobbee, Adriaan G. Holleboom, Manuel Castro Cabezas

**Affiliations:** ^1^ Department of Internal Medicine Franciscus Gasthuis & Vlietland Rotterdam the Netherlands; ^2^ Department of Internal Medicine and Endocrinology Erasmus MC Rotterdam the Netherlands; ^3^ Julius Clinical Zeist the Netherlands; ^4^ Department of Gastroenterology and Hepatology Franciscus Gasthuis & Vlietland Rotterdam the Netherlands; ^5^ Department of Gastroenterology and Hepatology Erasmus MC Rotterdam the Netherlands; ^6^ Department of Gastroenterology and Hepatology LUMC Leiden the Netherlands; ^7^ Department of Internal Medicine Amsterdam UMC Amsterdam the Netherlands

**Keywords:** MASH, MASLD, NAFLD, NASH, pharmacotherapy, T2DM

## Abstract

**Background:**

Metabolic dysfunction‐associated steatotic liver disease (MASLD) is closely related to type 2 diabetes mellitus (T2DM) through a common root in insulin resistance. The more severe stage, metabolic dysfunction‐associated steatohepatitis (MASH), increases the risk for cardiovascular complications, liver cirrhosis and hepatocellular carcinoma. Several trials investigating established antidiabetic‐drugs in patients with T2DM and MASLD have yielded promising results. Therefore, we aimed to systematically review the effect of T2DM‐drug treatment on MALSD parameters.

**Methods:**

Medical databases were searched until January 2025 for controlled trials in patients with T2DM and MASLD/MASH. Studies that evaluated the effect of T2DM‐medication on the severity of MASLD/MASH in T2DM patients were included. The quality of the studies was assessed by three independent reviewers using a set of Cochrane risk‐of‐bias tools.

**Results:**

Of 1748 references, 117 studies fulfilled the inclusion‐criteria and were assessed for eligibility in full‐text. Fifty‐two articles were included. Data included a total of 64.708 patients and study populations ranged from 9 to 50.742. Heterogeneity in study‐design and analysis hampered the comparability of the results. Most evidence was present for GLP‐1 receptor agonists, SGLT2‐inhibitors and PPAR‐γ‐agonists for regression of liver fibrosis and MASH.

**Conclusion:**

Studies on the value of T2DM‐drug treatment in the improvement of MASLD vary significantly in study design, size and quality. GLP‐1 receptor agonists, PPAR‐γ‐agonists, SGLT2‐inhibitors may all be preferred pharmacological interventions for patients with MASLD/MASH and T2DM. Newer agents like dual GLP‐1/GIP or triple GLP‐1/GIP/Glucagon agonists will likely play an important role in the treatment of MASLD/MASH in the near future.

## Graphical abstract

Pharmacological treatment options for metabolic dysfunction‐associated steatotic liver disease in patients with type 2 diabetes mellitus: a systematic review. For this systematic review medical databases were searched for studies that evaluated the effect of diabetes‐medication on the severity of metabolic dysfunction‐associated steatotic liver disease in type 2 diabetes mellitus (T2DM) patients. Of 1748 references, fifty‐two articles were included. Data included a total of 64.708 patients. Included studies vary significantly in study design, size and quality. GLP‐1 receptor agonists, PPAR‐γ‐agonists, SGLT2‐inhibitors and perhaps the newer GIP/GLP‐1 receptor agonists may all be preferred pharmacological interventions for patients with MASLD and T2DM.

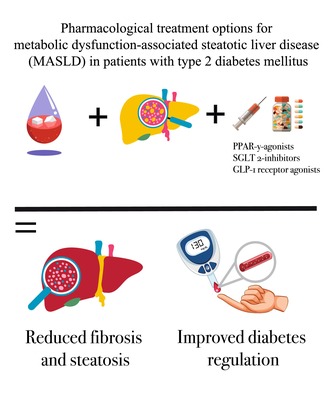

## 1 INTRODUCTION

Metabolic dysfunction‐associated steatotic liver disease is defined as an accumulation of lipids in more than 5% of the parenchymal cells of the liver in absence of excessive alcohol consumption, drug use or other diseases that can induce steatosis.^1^ Intrahepatic fat accumulation may cause inflammation, ballooning of the hepatocytes and fibrosis, which then is known as metabolic dysfunction‐associated steatohepatitis (MASH).^2^ This condition may lead to liver cirrhosis followed by liver failure or hepatocellular carcinoma.^3^ MASLD has been associated with obesity, metabolic syndrome and type 2 diabetes (T2DM). It may not be surprising that international studies suggest that over 60% of T2DM patients have MASLD.^4^

All of these metabolic diseases are closely linked to higher cardiovascular and liver‐related mortality which is a worldwide growing problem.^5^ MASLD on itself has also been associated with cardiovascular disease.^6^, ^7^ The prevalence of MASLD across the globe has been estimated to be close to 30%.^2^, ^8^ Despite these large numbers, there is a lack of awareness in diagnosing and staging MASLD in T2DM patients, even though early diagnosis and treatment could prevent or even reverse progression.^9^

Insulin resistance plays a crucial role in the development of MASLD, resulting in less efficient adipose tissue inhibition of lipolysis leading to an increased release of free fatty acids (FFAs) in the circulation.^10^ In addition, insulin resistance is associated with elevated concentrations of circulating triglyceride rich lipoproteins, the FFAs generated during lipolysis of these lipoproteins contribute significantly to hepatic steatosis.^11^ Finally, insulin resistance upregulates hepatic de novo lipogenesis.^12^ All these mechanisms closely related to insulin resistance lead to hepatic lipid accumulation.^10^ The accumulation of lipids and insulin resistance may lead to oxidative stress and activation of inflammatory pathways in the liver, resulting in cellular damage and finally fibrosis.^13^, ^14^

Various interventions such as lifestyle modifications, pharmacological treatment and bariatric surgery are currently being investigated in relation to MASLD/MASH.^15^, ^16^, ^17^ Weight reduction and dietary changes have shown to be effective in the treatment of MASLD, although this has not been established with internationally accepted criteria as those required for pharmacological interventions. Multiple drugs used in the treatment of T2DM such as sodium‐glucose cotransporter 2 (SGLT)‐inhibitors and glucagon‐like peptide 1 (GLP1) receptor agonists may have a beneficial effect on MASLD/MASH, while other drugs may have a negative effect due to increase in body weight like sulfonylurea derivatives (SU) and insulin.^17^ Several trials including T2DM treatments in subjects with MASLD have described promising results and large phase 3 studies are underway. The aim of this paper was to systematically review existing evidence for beneficial effects on MASLD by T2DM drug treatment in order to provide clinicians up to date information on which to base their treatment choices.

## 2 METHODS

This systematic literature review was conducted in accordance with the Preferred Reporting Items for Systematic Reviews and Meta‐analysis (PRISMA) guidelines and the Cochrane review handbook.^18^

### 2.1 Search Strategy

We conducted an extensive literature search to identify all studies evaluating T2DM medication in subjects with MASLD using the MEDLINE (Pubmed) electronic database, the Cochrane Library and CINAHL database. We included articles published until January 2025 and set a restriction on English language. Our search strategy included the terms: Metabolic dysfunction‐associated steatotic liver disease, “MASH”, “Non‐alcoholic Fatty Liver Disease”, “Nonalcoholic Steatohepatitis”, “Diabetes Mellitus”, “Hypoglycemic Agents” and “Pharmacotherapy”. The full search strategy has been included in Data S1. Additionally, reference lists were manually checked to identify potentially eligible studies.

### 2.2 Study selection

Eligible studies were (randomized) controlled trials or non‐randomized single arm studies conducted in adult patients with MASLD and T2DM on pharmacological diabetes treatment. We excluded studies that reported inclusion of patients with a history of liver transplantation, diagnosed with other liver diseases or with secondary causes of steatosis (e.g. viral hepatitis, autoimmune hepatitis, hepatocellular carcinoma, alcoholic fatty liver disease), type 1 diabetes or other types of diabetes and pregnant individuals. The primary outcome of interest was reduction in hepatic steatosis based on either imaging or biopsy. Secondary outcomes of interest included changes in MASLD (assessed by biomarkers), hepatic fibrosis (assessed by liver biopsy, imaging or biomarkers) and change in HbA1c, weight and liver enzymes. Review papers were excluded. Studies were reviewed and included by the authors independently (LK, LM, AB) and discrepancies were resolved through discussion.

### 2.3 Data extraction

Data from included studies was extracted using standardized templates. We registered study characteristics, interventions, measurement method, patient population characteristics and outcomes measured by imaging, scores or histology.

### 2.4 Quality assessment

The included studies were assessed for quality using Cochrane's Handbook Risk of Bias Tool by the authors. The Revised Cochrane risk‐of‐bias tool for randomized trials (RoB2) was used for the included randomized clinical trials. This tool focuses on different domains of bias (outcome data, randomization process, intervention and reporting) formulating a conclusion about methodological quality of the study evaluated.^18^ Single arm studies were assessed on risk of bias using the Risk of Bias in Non‐randomized studies of interventions (ROBINS‐I) assessment tools.^19^

### 2.5 Data analysis

Reduction of MASH was defined as improvement in hepatic steatosis based on imaging or biopsy in the measuring scale used in the reported article. Improvement of secondary outcomes was defined as improvement (change) of the assessed biomarkers. Improvement of hepatic fibrosis was defined as an improvement in the ratio, score or index as used in the specific study or as non‐worsening of the fibrosis. Total primary and secondary results on MASH improvement were summarized in a narrative overview. Due to expected heterogeneity in study design and study population pooled effect estimates were not calculated.

## 3 RESULTS

### 3.1 Study selection

**FIGURE 1:**
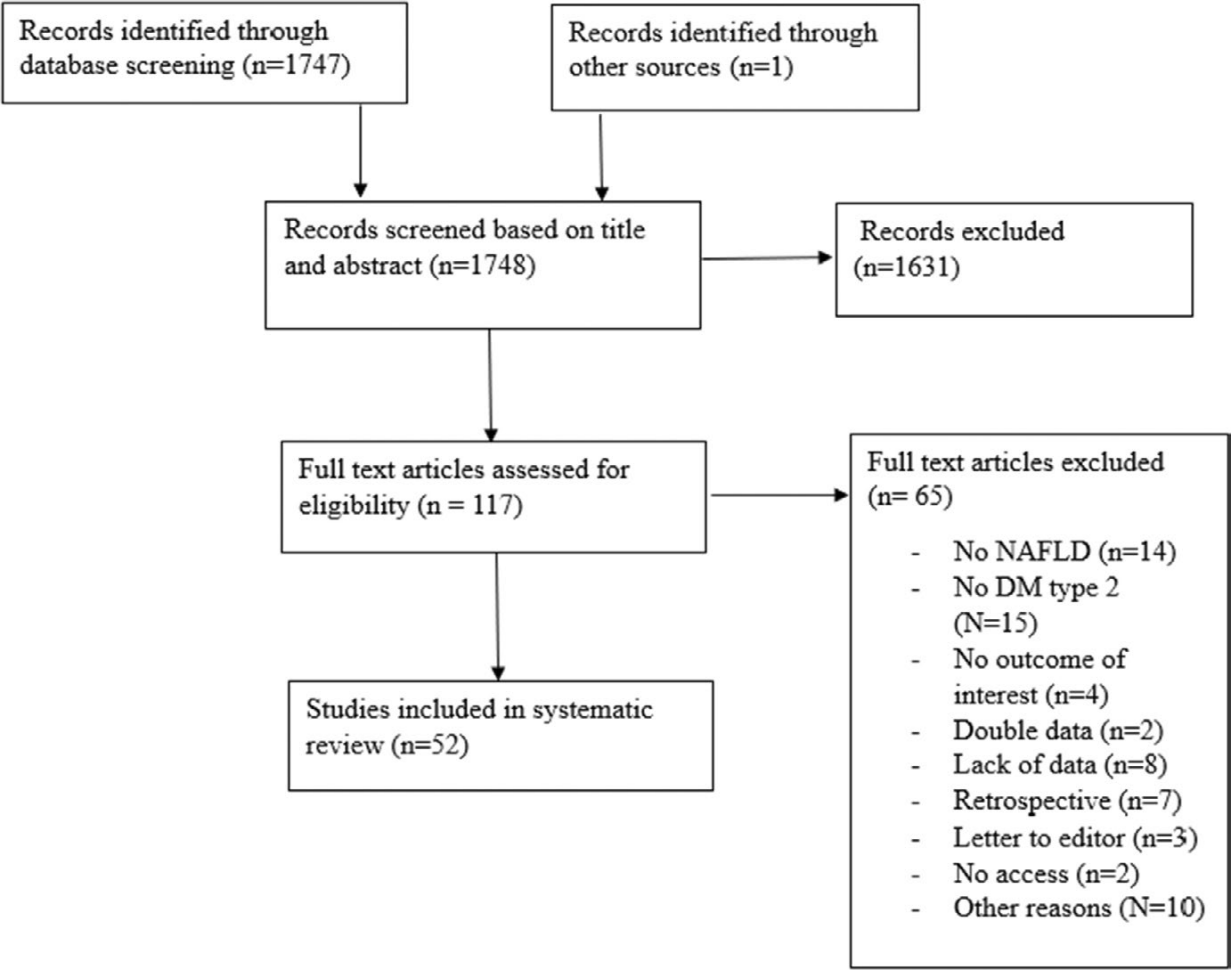
Flowchart summarizing the literature search and study selection process. DM, diabetic Mellitus; NAFLD, non‐alcoholic fatty liver disease.

In total 1748 articles were screened for eligibility. After checking for duplicates, articles were screened on abstract only (Figure 1). Most articles were excluded on subject and research design. Reviews and in‐vitro experiments were excluded. 117 articles were screened full text. After detailed review, 52 full‐text papers fulfilled the inclusion criteria. One trial included both, patients with and without T2DM and reported combined outcome measurements.^20^ Two trials reported on the same cohort but used two different interventions.^21^, ^22^ Another two trials used the same cohort, but the second trial combined the treatment of the first trial and did a follow up of another 24 weeks.^23^, ^24^

### 3.2 Methodological quality

The three reviewers agreed on 52 articles until January 2025. All 52 studies fulfilled the mandatory criteria. Median quality was moderate (as shown in Data S1). Interestingly, different studies were not designed to investigate the research question. D2 and D5 in the ROBINS‐2 assessment were therefore scored ‘some concerns.’ In the studies evaluated with ROBINS D1 and D6 were scored moderate, due to differences in trial design.

### 3.3 Overall study characteristics

**FIGURE 2:**
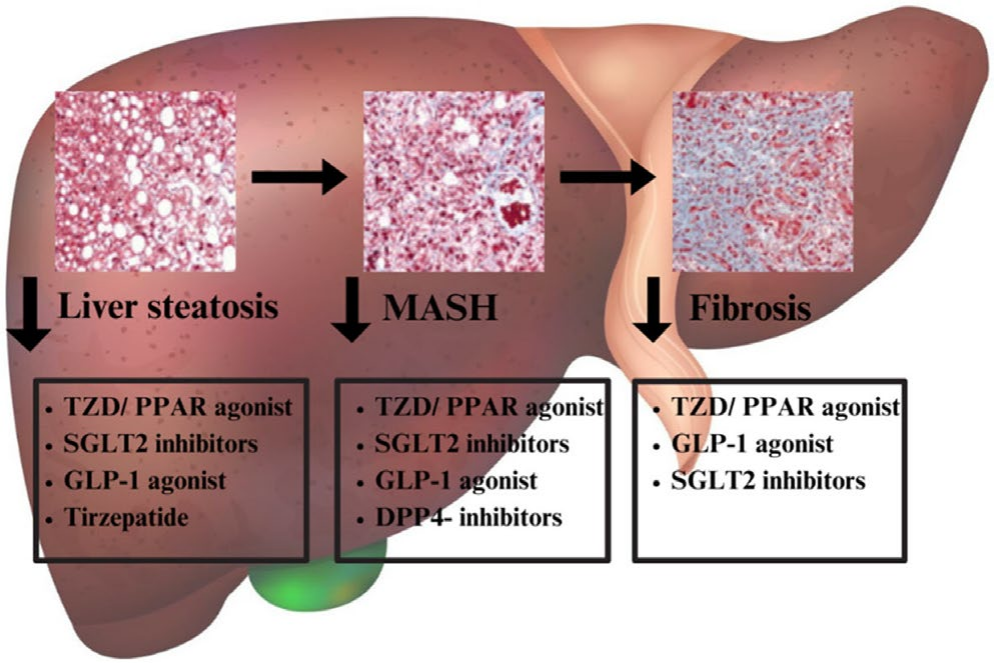
Different agents and their effects on steatosis, MASH or fibrosis. Overview of the different treatment modalities and where they exert their function on the different stages of metabolic dysfunction‐associated liver disease. The TZD derivates/ PPAR agonists, SGLT2 inhibitors, GLP‐1 agonist seem to improve liver steatosis as well as MASH and fibrosis, whereas Tirzepatide improves steatosis and DDP4‐inhibiors improve MASH. Legend; TZD, Thiazolidinedione derivates; PPAR, Peroxisome proliferator‐activated receptor, DPP4, dipeptidyl peptidase‐4; SGLT‐2, sodium‐glucose co‐transporter‐2; GLP1, glucagon‐like peptide‐1.

The number of included patients per study ranged from 9 to 50.742 and most patients were male. Data was collected from 64.708 patients in total. Patients differed in previous treatments and on‐treatment medication. Mostly, Fibroscan™, HR‐MRI, CT and biopsy were used to evaluate the degree of hepatic steatosis. The duration of the treatment periods ranged from 5 to 125 weeks. Detailed baseline characteristics are reported in Table 1. Outcomes of the different treatment modalities are reported in Tables 2 and 3. An overview of the different agents and their results is reported in Table 4. Figure 2 depicts a schematic picture of the various stages of disease progression where the different agents act to mitigate progression.

**TABLE 1:** Baseline characteristics of included studies.

Author, Year, Reference	Study type	Groups	Dosage	Patients (n)	Study length (weeks)	Female (%)	Mean BMI (kg/m^2^)	Diabetes duration (years)	Previous treatment	HbA1 (%)	Histology diagnose NASH (%)	Imaging	AST (U/L)	ALT (U/L)
TZD														
Lee Y 2017^26^	Non‐randomized, open label, single arm	Lobeglitazone	0.5 mg qd	43	24	35	27.5	‐	Drug‐free/naive/metformin	7.4	‐	Fibroscan	31.7	43.7
Cusi K 2016^27^	Single‐center, parallel‐group, randomized, placebo‐controlled study	A: pioglitazone B: placebo	45 mg qd	50 (24^#^) 51 (28^#^)	72	28 31	34.3 34.5	‐	Metformin, sulfonylureas, insulin	6.3 6.4	42 45	H‐MRS	47 43	62 57
Omer Z 2010^28^	Randomized, single‐center study	A:metformin B:rosiglitazone C:metformin and rosiglitazone	1700 mg/d 4 mg/d 1700 mg/d, 4 mg/d	22 20 22	48	32 55 50	30.8 28.4 32.5	‐	‐	5.8 6.0 6.9	100 100 100	‐	46.1 53.8 51.4	63.1 64.9 72.5
Belfort R 2006^29^	Randomized, double‐blinded, placebo‐controlled study	A: pioglitazone B: placebo	45 mg qd ‐	26 21	24	54 67	33.5 32.9	‐	‐	6.2 6.2	100 100	H‐MRS	47 42	67 61
Bril F 2018^30^	Prospective randomized study	A: pioglitazone B: placebo	45 mg qd ‐	52	72	29	34.4	‐	‐	6.9	100	H‐MRS	53	71
TZD/SGLT‐2 inhibitor														
Hooshmand Gharabagh L 2024^32^	Open label, randomized clinical trial	A: min 1500 metformin + empagliflozin B: min 1500 mg metformin + pioglitazone	A:10 mg qd B: 30 mg qd	32 30	24	43.3 40.0	31.23 28.48	‐	No SLGT2‐i, thiazolidinedione, stable dose of metformin for 3 months	8.73 8.82	‐	Ultrasound +fibroscan	22.90 20.26	27.60 24.73
Yoneda M 2021^23^	Open‐label, prospective, single center, randomized clinical trial	A: tofogliflozin B: pioglitazone	20 mg qd 15‐30 mg qd	21 19	24	38.1 57.9	29.4 30.8	‐	No other SGLT2, thiazolidinedione, insulin, GLP‐1 agonist or Vitamin E	7.22 7.06	‐	MRI‐PDFF	54.0 64.0	84.0 79.3
Yoneda M 2022^24^	Open‐label, prospective, single center, randomized clinical trial	A: tofogliflozin B: pioglitazone, followed by combination therapy	20 mg qd 15‐30 mg qd	20 12	48	40.0 50.0	29.6 31.5	‐	Other SGLT2, thiazolidinedione, insulin, GLP‐1 agonist	7.24 7.33	‐	MRI‐PDFF	53.0 70.0	82.2 82.2
Ito D 2017^31^	Randomized open‐label, active‐controlled trial	A: pioglitazone B: ipragliflozin	30 mg qd 50 mg qd	34 32	24	47 56	29.9 30.7	9.5 8.7	Metformin/DPP4‐inhibitor/sulfonylurea/insulin	8.3 8.5	‐	CT‐scan	43.3 39.7	53.1 57.4
Cho 2021^32^	Randomized, open‐label, parallel group trial	A: pioglitazone B: dapagliflazone after pioglitazone	30 mg qd 5 mg qd	26 27	24	50 44.4	‐ ‐	‐ ‐	Pioglitazone, sulfonylurea, insulin, DDP4, glinide	6.9 6.8	‐	‐	23.6 23.0	23.7 23.1
Kinoshita T 2020^33^	Prospective randomized open label study	A: dapagliflozine B: glimepiride C: pioglitazone	5 mg qd 0.9 mg qd^$^ 17.3 mg qd^$^	32 33 33	28	53.1 54.5 54.5	29.5 28.4 28.7	6.6 7.2 7.9	Glinide, DPP4, metformin, GLP‐1	7.4 7.6 7.4	‐	CT‐scan	38.8 32.3 34.1	50.3 45.3 46.1
Khaliq A 2024^43^	Prospective, randomized, double‐blind placebo‐contolled interventional stdy	A: Ertugliflozin B: pioglitzone C: placebo	15 mg qd 30 mg qd	65 65 65	24	16.6 16.6 21.6	31.8 30.7 30.1	‐	Not mentioned	7.2 7.4 7.0	‐	Ultrasound	98.5 90.0 95.5	86.6 96 95
Aghajanpoor M 2024^42^	Randomized clinical trial	A: pioglitazone B: pioglitazone + empagliflozin	15‐30 mg qd 15‐30 mg qd + 10 mg	42 43	24	52.4 48.8	25.6 29.4	‐	No pioglitazone, vitamin E, empagliflozin	7.75 10.05	‐	Ultrasound	26.8 26.9	30 32
TZD/DPP‐4 inhibitor														
Han E 2022^40^	Double‐blinde, active‐controlled, randomized, phase IV clinical trial	A: evogliptin: pioglitazone	5 mg qd 15 mg qd	25 26	24	20.0 50.0	28.76 28.46	5.08 4.12	Metformin	7.16 7.19	‐	MRI‐PDFF	44.00 41.88	58.52 66.00
GLP‐1‐agonist														
Guo W 2020^14^	Open label, prospective randomized placebo‐controlled single center study	A: Llraglutide B: insulin glargine C: placebo	1.8 mg qd >10 IU qd	31 30 30	26	48 40 33	29.2 28.3 28.6	‐	Metformin	7.5 7.4 7.4	‐	H‐MRS	29.6 27.9 28.1	33.2 31.5 30.5
Liu L 2020^16^	Open‐label, randomized, controlled, parallel‐group, multicentre clinical trial	A: exenatide bid B: insulin glargine *	10 μg bid	35 36	24	46 47	28.5 27.8	‐	‐	8.32 8.58	‐	H‐MRS	31.3 25.1	42.7 32.8
Newsome PN 2021^17^	Double‐blind, randomized, placebo controlled	A: semaglutide B: semaglutide C: semaglutide D: placebo	0.1 mg qd 0.2 mg qd 0.4 mg qd	80 78 82 80	72^●^	64 67 57 55	36.1 35.6 35.2 36.1	‐	‐	7.4 7.2 7.2 7.3	100 100 100 100	Fibroscan	44 43 44 42	55 53 54 55
Feng W 2017^18^	open‐label prospective randomized trial using a parallel design	A: liraglutide B: gliclazide C: metformin	1.8 mg qd 120 mg qd 1000 mg bid	29 29 29	24	28 31 34	28.1 27.9 26.8	‐	≥ 3 months drug naivety	8.91 9.03 9.36	‐	Ultrasound	31.22 28.45 34.09	49.73 44.99 51.01
Armstrong MJ 2016^15^	multicenter, double‐blindrandomized, study	A: liraglutide B: placebo	1.8 mg qd	26 26	48^●^	31 50	34.2 37.7	‐	metformin, sulfonylurea	5.9 6.0	26 26	‐	51 51	77 66
Eguchi Y 2015^19^	open label non‐randomized	A: liraglutide	0.9 mg qd	19	24	‐	31.6	‐	‐	6.5	100	CT‐scan	46.9	59.7
Shao N 2014^20^	open‐label randomized	A: exenatide and glargine insulin* B: insulin glargine and insulin aspart *	10 μg bid	30 30	12	50 53	30.6 30.29	‐ ‐	‐	7.68 7.59	‐ ‐	Ultrasound	125 122	170 164
Fan H 2013^21^	Open label	A: exenatide B: metformin	10 μg bid 2 g/d	49 68	12	43 44	28 27	‐		8.14 8.09	‐	Ultrasound	35.9 34.3	65.7 65.8
Kuchay 2020^22^	Open label, randomized controlled trial	A: dulaglutide B: control	1.5 mg qw standard care	27 25	24	28 31	29.6 29.9	4.9 5.7	metformine, DDP4, sulfonylurea	8.4 8.4	‐	MRI‐PDFF/Fibroscan	49.9 46.1	70.1 68.1
Jiang X 2024^30^	Randomized, double blind, controlled trial	A: metformin B: metformin + exenatide	2d 500 mg qd 2d 500 mg + 2d 5mcg qd	64 64	24	42.19 39.06	25.41 25.44	‐	‐	‐	‐	‐	60.93 61.05	59.36 59.84
Volpe S 2022^71^	Prospective, single‐arm, real life study	Semaglutide	0.25 up to 1 mg qw	48	52	45.8	38.8	6	metformin, and no GLP‐1a, SGLT2i, insulin or pioglitazone	7.0	‐	ultrasound	28.2	43.7
Parker V 2023^70^	Two‐part, randomized phase 2a trial	A: cotadutide B: placebo C: liraglutide	100 – 300mcg qd placebo part B: cotadutide 30–300 mcg qd B: placebo C: liraglutide 0.6–1.8 mg qd	9 11 10	5	33.0 18.0 40.0	31.9 30.8 30.2	8.1 7.0 9.5	‐	6.8 7.1 6.8	‐	MRI‐PDFF	21.0 21.0 17.5	26.9 26.9 23.4
Arai T 2024^72^	Single‐arm, multicentre, preospective study	A: oral semaglutide	A: 3, 7 or 14 mg qw	80	48	57	29.5	‐	No initiation of antidiabetic/ antidyslipidaemic medication 12 weeks before start trial	6.9	Biopsy proven or ultrasound proven	Biopsy proven or ultrasound proven	42	62
TZD/GLP‐1														
Zhang LY 2020^24^	open label, prospective, dubbel‐blind, randomized	A; liraglutide B: pioglitazone	1.2 mg qd 30 mg qd	30 30	24	57 50	27.6 27.1	‐	metformin	8.1 7.1	‐	H‐MRS	3.5^∆^ 3.5^∆^	3.4^∆^ 3.6^∆^
DDP4‐inhibitor/GLP‐1‐agonist														
Yan J 2019^25^	Open‐label, active‐Controlled, parallel‐group multicenter study	A: liraglutide B: sitagliptin C: Insulin glargine	1.8 mg qd 100 mg qd >0.2/kg/d	24 27 24	26	29 22 42	30.1 29.7 29.6	3.3 4.3 5.8	metformin	7.8 7.6 7.7	‐	MRI‐PDFF	31.1 34.4 33.2	43.2 46.0 39.5
SGLT2‐inhibitor														
Inoue M 2019^34^	Pilot, prospective, non‐randomized, open‐label single‐arm study	Canagliflozin	100 mg qd	20	52	45	31.5	7.4	Insulin/sulfonylurea/thiazolidine/biguanide/DPP4 inhibitors	8.7	‐	BIA/MRI	52	80
Shimizu M 2019^35^	Prospective, randomized, open‐label, blinded endpoint study	A: dapagliflozin B: control	5 mg qd Standard care	33 24	24	42 38	27.6 28.3	‐	Oral antidiabetic agents 1–3/ insulin	8 7.7	‐	BIA/FibroScan	28.0 26.0	38.0 33.0
Eriksson JW 2018^36^	Randomized placebo‐controlled double‐blind double‐dummy four‐armed parallel group trial	A: dapagliflozin B: OM‐3CA C: dapagliflozin and OM‐3CA D: Placebo	10 mg qd 4 g qd 10 mg, 4 g qd	19 15 20 19	12	24 45 32 19	30.5 33.0 31.1 30.3	6.7 6.3 8.5 6.5	Metformin/sulfonylurea/naïve	7.38 7.38 7.5 7.44	‐	MRI‐PDFF	31 31 30 29	40 38 37 34
Kuchay MS 2018^23^	Prospective, open‐label, randomized clinical study	A: Empagliflozin B: control	10 mg qd standard care	22 20	20	41.1 40.0	30.0 29.4	6.6 6.8	Metformin/DPP4‐inhibitors/sulfonylureas/insulin	9.0 9.1	‐	MRI‐PDFF	44.6 45.3	64.3 65.3
Lai LL 2020^42^	Single‐arm, open‐label, pilot study	A: empagliflozin	25 mg qd	9	24	65.6	29.8	17	Others drugs than SGLT2‐inhibitors/TZD/GLP1‐agonist	7.1	100	HepaFat–scan (MRI)	29	37
Sumida Y 2019^37^	Prospective, single‐arm trial	A: luseogliflozin	2.5 mg qd	40	24	30	27.76	8.9	Metformin/αgi/dpp/dpp4‐inhibitors/none	7.29	‐	MRI‐HFF	40.7	54.7
Tobita H 2017^57^	Single‐arm non‐randomized, open‐label study	Dapagliflozin	5 mg qd	16	24	45.0	31	‐	DPP4i,^14^ SU^1^	7.4	100	‐	52.0	59.0
Hussain M 2021^59^	Randomized controlled trial	A: dapagliflozin B: placebo + life style modifications	5‐10 mg qd placebo	75 75	12	37.0 34.5	29.5 31.5	‐	Glimepiride	7.5 8.2	‐	Ultrasound	74 71	69 68
Shi M 2023^31^	Prospective, open‐label, randomized controlled trial	A: dapagliflozin + metformin B: metformin + other treatment	10 mg qd	42 42	24	32.5 28.9	31.1 30.4	‐	Metformin	8.38 8.66	‐	MRI‐PDFF	26.4 27.8	39.6 44.4
Phrueksotsai S 2021^61^	Double‐blinded, placebo‐controlled, randomized, single‐center study	A: dapagliflozin B: placebo	10 mg qd placebo	20 20	12	72.2 65.0	29.6 28.8	4.5 5.5	stable dose of oral medication for at least 12 weeks, no use of weight loss medication or vitamin E	8.2 7.8	‐	Non‐contrast CT‐abdomen	25.5 22.0	35.0 27.0
Takahashi H 2022^51^	Multicenter, open‐label, randomized controlled trial	A: ipragliflozin B: lifestyle modifications and diet, antidiabetic drugs (exc. SGLT2s, pioglitazone, GLP1‐agonists or insulin)	A: 50 mg qd	27 28	72	37.5 46.2	29.9 28.8	‐	No SGLT2‐i, pioglitazone, GLP1‐agonists or insulin	6.5 6.8	100	‐	43.5 41.5	57.0 52.0
Borisov A 2023^60^	Post‐hoc analysis of 2 large double‐blind randomized controlled trials	A: canagliflozin B: placebo	100 or 300 mg qd placebo	5787 4344	125	35.8	‐	13.5	Other diabetic medication	8.2	‐	‐	23.0	26.0
Cusi K 2019^62^	Double‐blind, parallel‐group, placebo‐controlled trial	A: canagliflozin B: placebo	300 mg qd placebo	26 30	24	38.0 30.0	32.2 31.0	‐	Metformin monotherapy of metformin + DPP4‐i with a stable dose for at least 12 weeks prior to screening	7.6 7.7	‐	H‐MRS	22.0 27.0	23.0 35.0
Kahl S 2020^58^	Randomized, parallel‐group, double blind phase 4 trial	A: empagliflozin B: placebo	25 mg qd placebo	42 42	24	31.0 31.0	32.1 32.4	3 3.5	No diabetic treatment or washout of one month	6.8 6.7	‐	H‐MRS	25.0 25.0	32.0 36.0
Bellanti F 2022^52^	Obervational pilat study	A: SGLT2‐i + metformin B: DPP‐4 or thiazolidinediones + metformin	‐	26 26	26	42.3 42.3	34.8 34.5	‐	Metformin monotherapy	9.24 8.73	‐	‐	48.5 54.7	49.6 65.0
Elhini S 2022^54^	Randomized, double‐blinded clinical study	A: empagliflozin B: ursodeoxycholic acid C: placebo	A: 25 mg qd B: 250 mg 2qd	89 87 80	26	66.25 68.75 68.75	32.57 33.52 33.90	‐	Use of sulfonylurea for at least 6 months	8.97 8.54 7.98	‐	MRI‐PDFF	29.5 33.39 25.85	28.75 31.60 26.05
DPP4‐inhibitor/SGT‐2 inhibitor														
Hiruma S 2023^47^	Randomized, controlled trial	A: Empagliflozin B: sitagliptin	10 mg qd 100 mg qd	23 21	12	26.1 31.6	30.6 28.6	4.6 3.3	Sulphonureum, glinides, a‐glucosidase inhibitors	7.1 7.2	100	H‐MRS	37.0 37.7	59.2 60.6
Kim J 2022^46^	Matched cohort	A: SGLT2i B: DPP4i	‐	25,371 25,371	104	46.22 55.51	27.37 27.38	5.20 5.17	other treatment not mentioned	‐	‐	‐	24.10 24.10	30.93 30.99
DPP4‐inhibitor														
Yilmaz Y 2012^38^	Open‐label, single‐arm observational pilot study	A: sitagliptin	100 mg qd	15	52	53.3	30.7	‐	no other DM drugs	6.7	100	‐	46	30
Cui J 2016^39^	Randomized, double‐blind, allocation‐concealed, placebo‐controlled trial	A: sitagliptin B: placebo	100 mg qd	25 25	24	48 68	31.9 31.7	‐	‐	6.1 6.2	‐	MRI‐PDFF	28.0 29.0	43.0 40.0
Komorizono 2020^40^	Randomized open blinded endpoint trial	A: linalgiptine and metformine B: metformine	5 mg qd, 750 mg qd 1500 mg bid or tid	24 25	52	58.3 64	29.7 27.9	‐	no other DM drugs	7.0 7.2	‐	CT‐HU/ ultrasound	35.5 33.1	50.9 41.1
Wang X 2022^29^	Prospective, single‐center, open‐label comparative study	A: control B: sitagliptin C: metformin D: metformin + sitagliptin	0 100 mg qd 3× 500 mg qd 100 mg + 3× 500 mg qd	14 17 17 20	24	42.8 41.2 47.1 55.0	24.06 25.41 26.46 26.07	‐	‐	8.08 7.93 8.60 7.83	0.0	HD‐MRI	‐21.76 19.25 22.00	‐ 26.81 24.00 25.45
GLP‐1/GIP														
Gastaldelli A 2022^66^	Randomized, open label, phase 3 trial	A: Tirzepatide–5 mg ‐10 mg ‐15 mg B: Insuline degludec	5 mg qd 10 mg qd 15 mg qd ‐	71 79 72 74	52	38 48 35 46	34.5 33.1 33.4 33.0	7.9 9.4 8.7 7.0	Metformin alone or with SGLT2‐i	8.27 8.41 8.15 8.14	‐	MRI‐PDFF	22.8 21.8 22.5 21.1	32.3 29.5 30.0 27.4

Abbreviations: αGI, α‐glucosidase inhibitors; AST, aspartate aminotransferase; ALT, alanine aminotransferase; BIA, bioelectrical impedance analysis; BMI, body mass index; CT, computed tomography; D, day; DPP4, dipeptidyl peptidase‐4; GLP1, glucagon‐like peptide‐1; HbA1c, haemoglobin A1c; H‐MRS, proton magnetic resonance spectroscopy; MRI‐HFF, magnetic resonance imaging hepatic fat fraction; MRI‐PDFF, magnetic resonance imaging‐estimated proton density fat fraction; OM‐3CA, n‐3 carboxylic acids; SGLT‐2, sodium‐glucose co‐transporter‐2; SU, sulphonylurea derivates; TZD, thiazolidinedione.

* Dose depends on glucose level.

^#^ Patients with diabetes type 2.

^●^ Baseline characteristics were given for whole study population.

^∆^ On natural logaritmic scale.

^$^ Mean final dose.

**TABLE 2:** Outcome of included studies.

Author, Year, Reference	Groups	Δ Weight (kg)	Δ HbA1 (%)	Δ AST (U/L)	Δ ALT (U/L)	Δ ALT (%)	Δ SAT (cm^2^)	Δ SAT (L)	Δ AT (cm^2^)	Δ VAT (L)	Δ VFA (cm^2^)	Δ IHF (%)	Δ CAP (dB/m)	Δ LSM (kPa)	Δ HFF (%)	Δ Liver PDFF (%)	Δ Liver fat content (%)	L/S ratio
TZD																		
Lee YH 2017^34^	Lobeglitazone	1.4*	−0.8*	−4.5*	13.3**	‐	‐	‐	‐	‐	‐	‐	15.6**	−0.5	‐	‐	‐	‐
Cusi K 2016^35^	A: pioglitazone B: placebo ∞	0.3^#^ −1.2	−0.4 −0.2	−18^#^ −5	‐35ₓ −13	‐	‐	‐	‐	‐	‐	‐	‐	‐	‐	‐	‐12ₓ −4	‐
Omer Z 2010^27^	A: metformin B: rosiglitazone C: metformin and rosiglitazone	‐	0.0 −0.2 0.0	‐	‐	‐	‐	‐	‐	‐	‐	‐	‐	‐	‐	‐	‐	‐
Belfort 2006^36^	A: pioglitazone B: placebo	2.5 −0.5	−0.7 **^#^ −0.1	−19**^#^ −9	−39**ₓ −21	‐	‐	‐	‐	‐	‐	‐	‐	‐	‐	‐	−12**ₓ 0	‐
Bril F 2018^37^	A: pioglitazone B: placebo	2.9^#^ −0.6	−0.9^#^ −0.3	−32^#^ −5	−50^#^ −17	‐	‐	‐	‐	‐	‐	‐	‐	‐	‐	‐	‐	‐
TZD/SGLT‐2 inhibitor																		
Hooshmand Gharabagh L 2024^32^	A: min 1500 mg metformin + empagliflozin B: min 1500 mg metformin + pioglitazone	−5.78**• 0.93*	−1.84**–1.79**	−8.05**–4.43**	−8.34*–7.33**	‐	‐	‐	‐	‐	‐	‐	‐	−1.27**–1.41**	‐	‐	‐	‐
Yoneda M 2021^23^	A: tofogliflozin B: pioglitazone	−2.83* 1.39*	−0.4*–0.69**	−15.8*–27.9*	−23.3*–33.8**	‐	‐	‐	‐	‐	‐	‐	‐	−0.14–0.34*	‐	−4.12* –7.54**	‐	‐
Yoneda M 2022^24^	A: tofogliflozin B: pioglitazone, followed by combination therapy	−3.25* 2.46* −0.79	−0.36* –0.73* –0.80**	−13.8*–31.2* –25.2**	−19.3*–34.0** 35.7**	‐	‐	‐	‐	‐	‐	‐	‐	−0.11–0.43* –0.40**	‐	−3.38*–5.56**–5.98**	‐	‐
Ito D 2017^38^	A: pioglitazone B: ipragliflozin	0.9* −2.3*^●^	−1.11* −0.94*	−11.6* −12.6*	−17.5* −20.0*	‐	‐	‐	‐	‐	−2.6 −26.1*●	‐	‐	‐	‐	‐	‐	0.21* 0.22*
Cho 2021^39^	A: pioglitazone B:dapagliflozine	0.5** −4.2**	0.2 0.2	−0.4 −1.6	−1.4 −1.8	‐	‐	‐	‐	‐	‐	‐	‐	‐	‐	‐	‐	‐
Kinoshita T 2020^33^	A:dapagliflozine B: glimepiride C: pioglitazon	−2.8* ^● $^ 1.4* 2.5*	−0.52* −0.30 −0.48*	−8.7* 0.3∆ −7.1*	−12.8* −1.8∆ −15.1	‐	‐	‐	‐	‐	−19.4*^●^^ 6.8 2.6	‐	‐	‐	‐	‐	‐	0.17*^∆’^ 0.03! 0.22*^∆^
Khaliq A 2024^43^	A: Ertugliflozin B: pioglitzone C: placebo	−11.66** ‐ ‐	−1.1*# −0.6 1.0	−53** −49.5* 4.0	52.2** −20.2* 5.2	‐	‐	‐	‐	‐	‐	‐	‐	‐	‐	‐	‐	‐
Aghajanpoor M 2024^42^	A: Pioglitazone B: pioglitazone + empagliflozin	‐	‐1.43 −1.54	−7.3 −6.4	−9.9 −7.8	‐	‐	‐	‐	‐	‐	‐	‐	‐	‐	‐	‐	‐
TZD/DPP‐4 inhibitor																		
Han E 2022^40^	A: evogliptin B: pioglitazone	−0.30∆ 2.50	−0.31 –0.48	−2.00∆–12.00	−6.0∆ –22.0	‐	0.25∆ 14.05	‐	‐	4.75 1.45	‐	‐	‐	‐	‐	‐	−1.69 −6.02**∆	‐
GLP‐1‐agonist																		
Guo W 2020^63^	A:liraglutide B: insulin glargine C: placebo	−5.1*^#∆^ −0.9 −0.6	−0.7 −0.5 −0.1	−5.3* −2.3 −1.8	−6.0*^#∆^ −1.8 0.0	‐	−36*^#∆^ −24.5*^#^ −7.1	‐	‐	−47*^#∆^ −16.6*^#^ −3.5	‐	‐	‐	‐	‐	‐	−6.3*^#^ −3.4 −0.1	‐
Liu L 2020^64^	A: exenatide B: insulin glargine	−5.0 **^∆^ −1.25	−2.28**^●^ −1.82**	−12.3**^∆^ ‐2.66	−21.5**^∆^ ‐7.66**	‐	−28.4**^∆^ 2.59	‐	‐	−43.5**∆ ‐8.3	‐	‐	‐	‐	‐	‐	−17.55** −10.49**	‐
Newsome PN 2021 ∞^20^	A: semaglutide B: semaglutide C: semaglutide D: placebo	−3.24 −6.01 −9.11 −1.91	−0.73^∞^ −0.98 −1.05 −0.02	−14.05 −15.19 −21.4 −5.76	−20.72 −21.32 −30.95 −11.22	‐	‐	‐	‐	‐	‐	‐	−2.36 −4.75 −3.82 2.14	‐	‐	‐	‐	‐
Feng W 2017^25^	A: liragutide B: gliclazide C: metformin	‐5.6** −0.37 −3.58**	−3.01** −2.56** −3.33**	−7.2** −4.73** ‐11.45**	−22.3** −11.63** −22.57**	‐	‐	‐	‐	‐	‐	−23.6**^∆^ −13.4** −16.69**	‐	‐	‐	‐	‐	‐
Armstrong MJ 2016^∞^ 69	A: liraglutide B: placebo	−5.3^#^ −0.6	−0.53^#^ 0.00	−15.8 −8.6	−26.6 −10.2	‐	‐	‐	‐	‐	‐	‐	‐	‐	‐	‐	‐	‐
Eguchi Y 2015^69^	A: liraglutide	‐	−0.6**	‐17.4*	−25.6 *	‐	‐	‐	‐	‐	−19.8*	‐	‐	‐	‐	‐	‐	0.12*
Shao N 2014^65^	A: exenatide and insulin glargine B: insulin glargine and insulin aspart	−3.3**^●^ −7.8**	−1.42 ** −1.31**	−92.9**^●^ −79.0*	−127**^●^ −96.6**	‐	‐	‐	‐	‐	‐	‐	‐	‐	‐	‐	‐	
Fan H 2013 27	A: exenatide B: metformin	−4.16^●^ −1.98	−0.91 −0.89	−7.89^∆^ −5.11	−27.3^∆^ −12.85	‐	‐	‐	‐	‐	‐	‐	‐	‐	‐	‐	‐	‐
Kuchay 2020^21^	A: dulglutide B: control	−4.3∆ −2.0	−1.6 −1.3	−16.6 −7.3	−26.4 −13.9	‐	‐	‐	‐	‐	‐	‐	‐	‐	‐	−5.8^∆^ −2.3	‐	‐
Jiang X 2024^30^	A: metformin B: metformin + exenatide	‐	‐	−10.66 ‐18.21•	−12.32 −19.23•	‐	‐	‐	‐	‐	‐	‐	‐	‐	‐	‐	‐	‐
Volpe S 2022^71^	Semaglutide	‐10.78*	‐	−8.9*	−15.8*	‐	‐	‐	‐	−1.6*	‐	‐	‐	‐	‐	‐	‐	‐
Parker V 2023^70^	A: cotadutide B: placebo C: liraglutide	−2.5 – ‐2.8	‐	‐	‐	‐	‐	‐	‐	‐		−4.1# ∆ ‐1.8	‐	‐	‐	‐	‐	‐
Arai T 2024^72^	Oral semaglutide	−4.0**	−0.9**	−15.0**	−27**	‐	‐	‐	‐	‐	‐	‐	−18.0*	−0.7*	‐	‐	‐	‐
TZD/GLP‐1																		
Zhang LY 2020^41^	A; liraglutide B: pioglitazone	−10.1*£ 1.1	−1* −0.6*	−0.4£ −0.2£	−0.4£ 0.0£	‐	‐	‐	‐	‐	‐	‐	‐	‐	‐	−4.0*∆ −1.5	‐	‐
DDP4‐inhibitor/GLP‐1‐agonist																		
Yan J 2019^48^	A: liraglutide B: sitagliptin C: Insulin glargine	−3.6*† −1.7* −1.2	−1.0** −1.0* −0.7*	−1.8 −8.7* −2.9	−5.2 −11.2* −0.8	‐	−28.6*† −9.4 18.6	‐	‐	−20.9*† −13.6* 9.5	‐	‐	‐	‐	‐	−4.0**† −3.8* −0.8	‐	‐
SGLT2‐inhibitor																		
Inoue M 2019^56^	Canagliflozin	−2.9**	−1 *	−9*	−21*	‐	‐	‐	‐	‐	‐	‐	‐	‐	−5.5*	‐	‐	‐
Shimizu M 2019^50^	A: dapagliflozin B: control	−2.9**∆ −0.6	−6.6** −7.49	−0.5* −2.4	−11.5** −1.0	‐	−11.2* 1.3	‐		−7.3** −5.7	‐	‐	−24.3*∆ 5.3	−1.48 0.45	‐	‐	‐	‐
Eriksson JW 2018^53^	A: dapagliflozin B: OM‐3CA C: dapagliflozin +OM‐3CA D: placebo	−2.44# −0.16 −2.16# −0.27	−0.63# 0.13 −0.45 −0.09	−4# 5 1 −1	−8# 6 0.06 −0.2	‐	‐	‐	‐	‐	‐	‐	‐	‐	‐	−2.23 −3.15 −3.15# −0.59	‐	‐
Kuchay MS 2018^22^	A: empagliflozin B: control	−3.3* −1.6*	−1.8** −2.0**	−8.4* −0.7	−14.6*∆ −3.7	‐	‐	‐	‐	‐	‐	‐	‐	‐	‐	−4.9**● −0.9	‐	‐
Lai LL 2020^55^	A: empagliflozin	‐	−0.5	−4	−8	‐	‐	‐	‐	‐	‐	‐	‐	‐	‐	‐	−7.8*	‐
Sumida Y 2019^49^	A: luseogliflozin	−1.43**	−0.29**	−8.8**	−12.3**	‐	‐	‐	‐	‐	‐	‐	‐	‐	‐	−5.8**	‐	‐
Tobita H 2017^57^	Dapagliflozin	−3.8*	−3.7*	−26*	−29*	‐	‐	‐		‐	‐	‐	‐	‐	‐	‐	‐	‐
Hussain M 2021^59^	A: dapagliflozin B: placebo + life style modifications	−6# –0.5	−3# –0.6	−27# –6	−17# 4	‐	‐	‐	‐	‐	‐	‐	‐	‐	‐	‐	‐	‐
Shi M 2023^31^	A: dapagliflozin + metformin B: metformin + other treatment	−5.6**• 0.3	−1.97** –2.03**	−8.4** –3.58	−12.09**• –4.07		‐	‐	‐	‐	‐	‐	‐	‐	‐	‐	−4.18**• 0.13	
Phrueksotsai S 2021^61^	A: dapagliflozin B: placebo	−2.3**ₓ –0.1	−1.3**# –0.2	−3.5* 0	−4.5*# –1	‐	‐	‐	‐	‐	‐	‐	‐	‐	‐	‐	‐	‐
Takahashi H 2022^51^	A: ipragliflozin B: lifestyle modifications, antidiabetic drugs (exc. SGLT2s, pioglitazone, GLP1‐agonists)	7.1 0.9	−0.5* –0.2	−10 5	−13 –0.5	‐	−21 0	‐	‐	‐	10 –2	‐	‐	‐	‐	‐	‐	‐
Borisov A 2023^60^	A: canagliflozin B: placebo	‐	‐	‐	‐	‐	‐	‐	‐	‐	‐	‐	‐	‐	‐	‐	‐	‐
Cusi K 2019^62^	A: canagliflozin B: placebo	−5.5# (%) –2.1 (%)	−0.7ₓ 0.1	‐	−3 –1		‐	‐	‐	‐	‐	−6.9# –3.8	‐	‐	‐	‐	‐	‐
Kahl S 2019^58^	A: empagliflozin B: placebo	−2.7ₓ –0.1	−0.06 0.13	‐	‐	−20 –13	‐	‐	‐	−0.251 0.0 39	‐	‐	‐	‐	‐	‐	−1.8# –0	‐
Elhini S 2022^54^	A: empagliflozin B: ursodeoxycholic acid C: placebo	‐	−1.72** −1.14** −0.61**	−11.50* −15.24* 3.40*	−13.00** −12.1** 4.35	‐	‐	‐	‐	‐	‐	‐	‐	‐	‐	‐	−8.73**∆ −5.71*** −1.99**	‐
DPP4‐inhibitor/SGLT‐2 inhibitor																		
Hiruma S 2023^47^	A: Empagliflozin B: sitagliptin	−1.8*∆ –0.2	−0.6** –0.6**	−7.3* –2.2	−13.3* –10.1	‐	‐	‐	‐	‐	‐	−5.2∆ –1.9	‐	‐	‐	‐	‐	‐
Kim J 2022^46^	A: SGLT2i B: DPP4i	‐	‐	−2.3• –0.8	−4.7 • –2.6	‐	‐	‐	‐	‐	‐	‐	‐	‐	‐	‐	‐	‐
DPP4‐inhibitor																		
Yilmaz Y 2012^44^	A: sitagliptin	‐	−0.2	−16*	−25*	‐	‐	‐	‐	‐	‐	‐	‐	‐	‐	‐	‐	‐
Cui J 2016^45^	A: sitagliptin B: placebo	0.2 −0.2	0 0.1	−1 −5	−9 −11.5	‐	‐	‐	‐	‐	‐	‐	‐	‐	‐	−8.4 −13.9	‐	‐
Komorizono 2020^28^	A: linagliptine and metformine B:metformine	−0.4 −1.6*	−0.2 −0.2	−1.1 −1.2	−0.2 −0.2	‐	‐	‐	‐	‐	‐	‐	‐	‐	‐	‐	‐	‐
Wang X 2022^29^	A: control B: sitagliptin C: metformin D: metformin + sitagliptin	‐ – – –	0.08–0.98* ^∆^! 0.22–0.79+	‐ 0.24! 1.95 –0.86	0.58! –2* –0.31	‐	‐	‐	‐	‐	‐	‐	‐	‐	‐	‐	−6.73 –19.77–36.16*–23.18	‐
GLP‐1/GIP																		
Gastaldelli A 2022^66^	A: Tirzepatide–5 mg –10 mg ‐15 mg B: Insuline degludec	‐	‐	‐	‐	‐	‐	−1.4** ^●^ ‐2.25** ^●^ −2.05** ^●^ 0.63*	‐	−1.10** ^●^ −1.53** ● −1.65** ● 0.38*	‐	‐	‐	‐	‐	‐	−6.35** ^∆^ −8.21** ● −7.78** ● −3.19**	‐

*Note*: Data are mean. *p <.05 for comparison before vs. after treatment within groups. **p <.001 comparison before vs. after treatment within groups.∆*p* <.05 are for comparison of change between groups (A vs. B). ‘*p* <.05 are for comparison of change between groups (A vs. C). !*p* <.05 are for comparison of change between groups (B vs. C); p <.05 are for comparison of change between groups (B vs. D). + *p* <.05 are for comparison of change between groups (C vs. D).^*p* <.001 are for comparison of change between groups (A vs. C). ●p <.001 are for comparison of change between groups (A vs. B). $*p* <.001 are for comparison of change between groups (B vs. C). ₓ *p* < 0.001 for comparison before vs. after treatment between groups (A or B or C vs. placebo). † *p* <.05 for comparison before vs. after treatment among groups. ∞ *p* values only between groups, data of diabetics and non‐diabetics combined. £logaritmic scale, #p <.05 for comparison before vs. after treatment between groups (A or B or C vs. placebo).

Abbreviations: AST, aspartate aminotransferase; ALT, alanine aminotransferase; CAP, controlled attenuation parameter; DPP4, dipeptidyl peptidase‐4; GLP1, glucagon‐like peptide‐1;HbA1c, haemoglobin A1c; HHF, hepatic fat fraction; IHCL, intrahepatic content of lipid; IHF, intrahepatic fat; LS, liver stiffness measurement; L/S, liver‐to‐spleen; OM‐3CA, n‐3 carboxylic acids; SAT, subcutaneous adipose tissue; SGLT‐2, sodium‐glucose co‐transporter‐2; TZD, thiazolidinedione; VAT, visceral adipose tissue; VFA, visceral fat area.

**TABLE 3:** Outcome of studies, non‐invasive and invasive measurements.

Author, Year, Reference	Groups	NFS	FIB‐4 index	NAFIC score	NASH score improvement (%)	Reversal rate Severity FL	Histology improvement in NAS	Resolution of NASH without worsening of fibrosis (%)	Improvement in liver fibrosis and no worsening of NASH (%)	Fibrosis improvement (%)
TZD										
Cusi K 2016^35^	A: pioglitazone B: placebo	‐	‐	‐	‐	‐	29%#∞ 9%	51# 19	‐	20 13
Omer Z 2010^27^	A: metformin B: rosiglitazoneC: metformin and rosiglitazone	‐	‐	‐	‐	‐	0.7 score ‐2.6 score* −3.9 score*	‐	‐	‐
Belfort R 2006^36^	A: pioglitazone B: placebo	‐	‐	‐	‐	‐	43%**#∞ 0%	‐	‐	46* 33
Bril F 2018^37^	A: pioglitazone B: placebo	‐	‐	‐	70*# 24	‐	‐	60*# 16	‐	−0.5^^#^ 0.2a
Ito D 2017^38^	A: pioglitazone B: ipragliflozin	‐	−0.16* −0.22*	‐	‐	‐	‐	‐	‐	‐
Cho 2021^39^	A: pioglitazone B: dapagliflozin	‐	−0.03 −0.17*#	‐	‐	‐	‐	‐	‐	‐
TZD/SGLT‐2 inhibitor										
Yoneda M 2021^23^	A: tofogliflozin B: pioglitazone	‐	−0.15 ‐0.38	‐	‐	‐	‐	‐	‐	‐
Khaliq A 2024^43^	A: Ertugliflozin B: pioglitazone C: placebo	‐	−0.78* −0.59* 0.10	‐	‐	‐	‐	‐	‐	‐
Aghajanpoor M 2024^42^	A: pioglitazone B: pioglitazone + empagliflozin	−0.24 −0.32’	−0.16 −0.19*	‐	‐	‐	‐	‐	‐	‐
*GLP‐1‐agonist*										
Liu L 2020^64^	A: exenatide B: insulin glargine	‐	−0.10* 0.13	‐	‐	‐	‐	‐	‐	‐
Newsome PN 2021^20^	A: semaglutide B: semaglutide C: semaglutide D: placebo	‐	‐	‐	‐	‐	‐	40.8# 37.3# 57.1∆ 18.0	40.8 31.4 40.8 26.0	
Armstrong MJ 2016^68^	A: liraglutide B: placebo	‐	‐	‐	‐	‐	74%∞ 64%	39# 9		26∞ 14
Eguchi Y 2015^69^	A: liraglutide	‐	0.2	‐	80%*	‐	70%*	‐	‐	60*
Shao N 2014^65^	A: exenatide and insulin glargine B: insulin glargine and insulin aspart	‐	‐	‐	‐	93.6** 66.7	‐	‐	‐	‐
Arai T 2024^72^	Oral semaglutide	‐	0.21**	‐	‐	‐	‐	‐	‐	‐
*DDP4‐inhibitor/GLP‐1‐agonist*										
Yan J 2019^48^	A: liraglutide B: sitagliptin C: Insulin glargine	0.03 −0.07 0	0 −0.12 0	‐	‐	‐	‐	‐	‐	‐
*SGLT2‐inhibitor*										
Inoue M 2019^56^	Canagliflozin	0.06	−0.05	‐	‐	‐	‐	‐	‐	‐
Shimizu M 2019^50^	A: dapagliflozin B: control	0.12 −0.29	−0.05 0.06	−1.0 0	‐	‐	‐	‐	‐	‐
Lai LL 2020^55^	A: empagliflozin	‐	‐	‐	‐	‐	‐	44†	‐	75†
Sumida Y 2019^49^	A: luseogliflozin	−0.01	−0.11	‐	‐	‐	‐	‐	‐	‐
Tobita H 2017^57^	Dapagliflozin	‐	−0.24	‐	‐	‐	‐	‐	‐	‐
Shi M 2023^31^	A: dapagliflozin + metformin B: metformin + other treatment	‐	−0.14*–0.06	‐	‐	‐	‐	‐	‐	‐
Takahashi H 2022^51^	A: ipragliflozin B: lifestyle modifications, antidiabetic drugs (exc. SGLT2s, pioglitazone, GLP1‐agonists)	‐	‐	‐	‐	‐	‐	66.7 27.3	‐	57.1∆ 16
Borisov A 2023^60^	A: canagliflozin B: placebo	−0.097# − 0.047	‐0.04 –0.06	‐	‐	‐	‐	‐	‐	‐
Elhini S 2022^54^	A: empagliflozin B: ursodeoxycholic acid C: placebo	−1.00** −1.11** 0.29	−0.34** −0.55* 0.06	‐	‐	‐	‐	‐	‐	‐
DPP4‐inhibitor/SGT‐2 inhibitor										
Hiruma S 2023^47^	A: Empagliflozin B: sitagliptin	‐	‐0.02 0.07	‐	‐	‐	‐	‐	‐	‐
DPP4‐inhibitor										
Yilmaz Y 2012^44^	A: sitagliptin	‐	‐	‐	‐	‐	−1.14 score*	‐	‐	−0.01
GLP‐1/ Glucagon receptor co‐agonist										

*Note*: aHistological score of fibrosis. #*p* < 0.05 for comparison before vs. after treatment between groups (A or B or C vs. placebo). †No *p*‐value available. ∞p values only between groups, data of diabetics and non‐diabetics combined. *p <.05 for comparison before vs. after treatment within groups. **p <.001 for comparison before vs. after treatment within groups. ≥2 point reduction in NAS without worsening fibrosis improvement.for comparison before vs. after treatment between groups (A or B). ∆*p* < 0.001 for comparison before vs. after treatment between groups (A or B or C vs. placebo).

Abbreviations: DPP4, dipeptidyl peptidase‐4; FIB4‐index, The Fibrosis‐4 index; FL, fatty liver; GLP1, glucagon‐like peptide‐1; NAFLD, non‐alcoholic fatty liver disease; NFS, NAFLD fibrosis score; NAS, nonalcoholic fatty liver disease activity score; NASH, non‐alcoholic steatosis hepatis; OM‐3CA, n‐3 carboxylic acids; SGLT‐2, sodium‐glucose co‐transporter‐2; TZD, thiazolidinedione. *p* < 0.05 *p* < 0.05.

**TABLE 4:** Outcome per agent/group.

Agent/Group	Number of studies	Change in weight	Change in liver enzymes	Reduction hepatic fat content (MRI)	Histology improvement in NAS	Histology improvement in fibrosis
Metformin	8	Variable results	Variable results	Significant decrease in fat content in one study in one study. No significant reduction in MASLD severity in the other studies.	No improvement	No improvement
Sulfonylurea derivates	2	Variable results	Variable results	One study showed improvement	No biopsy proven studies	No biopsy proven studies
Thiazolidinedione derivates and PPAR agonist	13	Weight gain in five studies	Variable results	Five studies showed significant reduction	Improvement in NAS in four studies	Improvement in fibrosis in one study
DPP4‐inhibitors	8	Variable results	Variable results	Significant reduction in one study, no reduction in two studies	One biopsy proven study that showed improvement in NAS score	No improvement in fibrosis, one biopsy proven study
SGLT2 inhibitors	25	Weight reduction in nineteen studies	Improvement of liver enzymes in nineteen studies	Significant improvement in fourteen studies	Two biopsy proven studies, both showed improvement	Two biopsy proven studies, both showed improvement
Insulin	5	No reduction	No reduction	Two studies showed change in fat content and two studies found no change	No biopsy proven studies	No biopsy proven studies
GLP1 receptor agonists	15	Weight reduction in eleven studies	Significant improvement in ten studies	Six studies looked into fat content and showed significant reduction	Three biopsy proven studies. Two studies showed improvement in NAS score	Three biopsy proven studies. One study showed improvement.
Tirzepatide	1	No information	No information	One study showed significant reduction	No biopsy proven studies	No biopsy proven studies

*Note*: This table describes the different treatment modalities and their effect on weight, liver enzymes, hepatic fat content, NAS score and fibrosis.

Abbreviations: DPP4, dipeptidyl peptidase‐4; SGLT‐2, sodium‐glucose co‐transporter‐2; GLP1, glucagon‐like peptide‐1; NAS, Nonalcoholic Fatty Liver Disease Activity Score; PPAR, Peroxisome proliferator‐activated receptor.

### 3.4 Reported effects per medication group

#### 3.4.1 Metformin

Six trials incorporated a treatment arm with metformin,^25^, ^26^, ^27^, ^28^, ^29^, ^30^ two trials used metformin plus another treatment as treatment arm.^31^, ^32^ Two trials used metformin as a reference group to evaluate the effect of GLP‐1 agonists,^25^, ^26^ one trial evaluated the effect of rosiglitazone versus metformin in combination with rosiglitazone or metformin only^27^ and one trial used metformin as a reference group to evaluate the effect of DPP4.^28^ One trial used liver biopsy as a reference.^27^

The study of Feng et al. showed a significant decrease in intrahepatic fat content (IHF) assessed by ultrasound in 29 patients.^25^ The other studies, including one biopsy proven study, did not show a significant reduction in MASLD severity.^26^, ^27^, ^28^ No change in fibrosis was detected.^27^ Variable results were found for change in Hb1Ac and weight.

#### 3.4.2 Sulfonylurea derivatives (SU)

SU were evaluated in two trials, which were part of a multiple arm trial.^25^, ^33^ The study of Kinoshita et al., which included three treatment arms of dapagliflozin, pioglitazone and glimepiride was negative concerning the effects of glimepiride on L/S and VFA ratios. In addition, no significant reduction in body weight, HbA1c or transaminases were detected. However, in the study of Feng et al. an improvement in IHF was found in all treatment arms, including the one with gliclazide.

#### 3.4.3 Thiazolidinedione (TZD) derivatives and peroxisome proliferator‐activated receptor (PPAR) agonists

In total 15 trials evaluated the effect of different TZD derivatives or PPAR agonists.^23^, ^24^, ^27^, ^32^, ^33^, ^34^, ^35^, ^36^, ^37^, ^38^, ^39^, ^40^, ^41^, ^42^, ^43^ Two trials included comparison with placebo, one trial with metformin (with or without a combination of rosiglitazone) and the others with liraglutide, ipragliflozin, dapagliflozin, empagliflozin, tofogliflozin, ertugliflozin and evogliptin.^23^, ^24^, ^27^, ^32^, ^35^, ^36^, ^37^, ^38^, ^39^, ^40^, ^41^, ^43^ Therapy was continued from 24 to 72 weeks. One single arm trial was included with a treatment arm of lobeglitazide.^34^ In the study of Kinoshita et al. three treatment arms were applied including two TZD's and one with a SGLT inhibitor.^33^ In four studies liver histology as a reference was used.^27^, ^35^, ^36^, ^37^ In the studies of Cusi et al. and Omer et al. also pre‐diabetic patients (or with an impaired glucose intolerance) were included.^27^, ^35^

The studies reporting histological endpoints showed an ≥2 reduction in non‐alcoholic fatty liver disease activity score without worsening of the fibrosis, significantly more compared to the placebo arm.^27^, ^35^, ^37^ In addition, in the study by Cusi et al. there was a significant reduction in fibrosis score.^35^ The study of Omer et al. showed a significant reduction of the histological NAFLD score in the rosiglitazone arm. No significant change was seen in fibrosis score.^27^ In the study of Belfort et al. a histological improvement in steatosis and inflammation was observed.^36^

In eight other studies, TZDs showed improvement of MASLD, Fibrosis‐4 index for liver fibrosis (FIB4) and controlled attenuation parameter (CAP) scores. In the study of Zhang et al. no significant difference was found in the hepatic fat content or liver enzymes. In this study pioglitazone was compared to liraglutide.^41^ In addition, in the study which compared dapagliflozin to pioglitazone no significant FIB4 reduction in the pioglitazone arm was found.^39^ The trial by Han et al. compared evogliptin and pioglitazone showing significant improvement in hepatic fat fraction with pioglitazone.^40^ The trial of Yoneda et al.comparing tofogliflozin and pioglitazone and the follow up trial which combined both therapies, also showed significant improvement in Liver PDFF with both therapies.^23^, ^24^

Interestingly, in the secondary outcomes no significant reduction in weight was seen and in six trials there was a significant weight gain (Table 2) in the treatment arm.^33^, ^34^, ^36^, ^37^, ^39^, ^43^ Also for other outcomes, for example in levels of transaminases and Hb1Ac, variable changes were reported. Ten trials reported a significant decrease of the Hb1Ac levels and four showed no significant change.^27^, ^39^, ^42^, ^43^ In three studies^33^, ^39^, ^41^ no change of transaminases was reported; in six studies a reduction was seen.^27^, ^36^, ^37^, ^38^, ^42^, ^43^

#### 3.4.4 SixDipeptidylpeptidase‐4(DPP4)‐inhibitors

Eight studies investigated the effect of DPP4‐inhibitors compared to placebo, metformin and insulin, liraglutide (GLP‐1) or SGLT2 inhibitors.^28^, ^29^, ^40^, ^44^, ^45^, ^46^, ^47^ (Table 2) The single arm study of Yilmaz et al. included histologically characterized steatotic liver disease.^44^ They reported a significant reduction in the NAFLD score without an improvement in the fibrosis grade. The two studies by Cui et al. and Komorizono et al. showed no significant improvement in measured hepatic fat content.^28^, ^45^ In the study of Yan et al. a significant reduction in MRI‐PDFF measurements were reported in the sitagliptin arm, but there was no difference in the liraglutide arm.^48^

#### 3.4.5 Sodium glucose cotransporter‐2 (SGLT2) inhibitors

Twenty‐five trials evaluated the use of SGLT2 inhibitors. Seven trials compared SGLT2 inhibitors to pioglitazone,^23^, ^24^, ^32^, ^38^, ^39^, ^42^, ^49^ seven included a control arm (diabetic medication)^22^, ^31^, ^46^, ^47^, ^50^, ^51^, ^52^ and four studies reported a multiple arm trial with pioglitazone, glimepiride, ursodeoxycholic acid or omega‐3 (n‐3) carboxylic acids (OM‐3CA) compared to placebo.^33^, ^43^, ^53^, ^54^ One trial combined empagliflozin with pioglitazone.^42^ Four single arm trials were included.^49^, ^55^, ^56^, ^57^ Five trials compared SGLT‐2 inhibitors with placebo.^58^, ^59^, ^60^, ^61^, ^62^ Therapy was continued for 12 to 125 weeks. Treatment arms differed in size from 9 to 50.742 patients. Only two single arm trials used histology, with biopsies pre‐and post‐treatment.^51^, ^55^

The study of Lai et al. showed a resolution of steatohepatitis in 44% of the patients treated with empagliflozin and improvement of fibrosis in 75%.^55^ Interestingly, this study did not show a significant reduction in the secondary outcome measurements. The study of Takahashi et al. found a significant reduction in steatohepatitis and an improvement in fibrosis in the ipragliflozin group.^51^

Different non‐invasive liver tests and imaging techniques (CAP, LSM, FIB4), liver fat content measurement with MRI and MRI‐proton density fat fraction (MRI‐PDFF) were used to study the effect of these compounds compared to standard care or placebo. Sixteen studies showed an improvement in MASLD severity compared to placebo or other trial arms (effects shown in Tables 2 and 3). In the single arm trial of Inoue et al. no significant reduction of the NAFLD score was detected (MRI).^56^ Three studies found no significant reduction of the Hb1Ac levels or transaminases.^39^, ^55^, ^58^

#### 3.4.6 Insulin

Although no placebo‐controlled trials were found evaluating the effect of insulin on MASH, five trials did use treatment arms with insulin.^48^, ^63^, ^64^, ^65^, ^66^ Insulin was compared to GLP1‐agonist, tirzepatide and DDP4‐inhibitors or in combination therapy. None of these studies included histological liver improvement.

In the study of Yan et al. no significant decrease in MRI‐PDFF or FIB4 score was found. In addition, the study of Shao et al. did not show a significant reduction in fatty liver content, body weight or transaminases.^48^, ^65^ The study of Guo et al. reported a significant decrease in intrahepatocellular lipids, but not in visceral adipose tissue or subcutaneous adipose tissue. In addition, no significant reductions in Hb1Ac weight or transaminases were found.^63^ The study of Gastaldelli et al. found improvement in liver fat content after treatment with insulin.^67^

#### 3.4.7 Glucagon like polypeptide (GLP‐1) receptor agonists

Fifteen RCTs were included which compared the use of GLP‐1 receptor agonists with insulin therapy, metformin, TZD, DPP4‐inhibitors or placebo.^20^, ^21^, ^25^, ^26^, ^30^, ^41^, ^48^, ^63^, ^64^, ^65^, ^68^, ^69^, ^70^, ^71^, ^72^ Three single arm trials were included.^69^, ^71^, ^72^ Therapy was continued for 5–72 weeks. Three studies used liver histology as reference standard.^20^, ^68^, ^69^ Patients with and without T2DM were included.^20^, ^68^

In the paper by Newsome et al. 40%–51% of the MASH improved significantly in the semaglutide arm but compared with placebo the difference was not significant for fibrosis.^20^ The study by Armstrong et al. showed improvement of MASH in 39% by liraglutide, but compared to placebo the difference was not significant. Fibrosis did not improve significantly in the liraglutide group, however, compared to placebo less patients showed worsening of fibrosis.^68^ In the single‐arm study by Eguchi et al. NAFLD scores were reduced significantly in 80% of patients treated with liraglutide and liver fibrosis improved significantly in 60%.^69^

Studies used different imaging endpoints to assess MASLD, but overall GLP‐1 receptor agonists had a significant effect on improved MASLD endpoints assessed by imaging. GLP‐1 receptor agonists had a significant effect on weight and HbA1c reduction. Liver enzymes improved significantly in most studies compared to the control group.

#### 3.4.8 Tirzepatide for T2DM and MASLD/MASH

The trial of Gastaldelli et al. compared the effect of tirzepatide to insulin on liversteatosis.^66^ This trial showed a significant reduction in liver steatosis, measured with MRI‐PDFF compared to insulin Degludec.

#### 3.4.9 Other possible candidates for the treatment of MASLD and MASH related liver fibrosis

Recently Resmetirom, a thyroid hormone receptor beta agonist that showed significant effects on MASH and fibrosis improvement, was approved for the treatment of MASLD/MASH.^73^, ^74^ FGF21 analogues have also shown promising results in fibrosis and MASH reduction.^75^ Advanced‐phase clinical trials investigate the effectiveness of FGF19 agonists, GLP/GIP dual agonists, Pan‐PPAR agonists and a triple hormone receptor agonist.^76^, ^77^, ^78^, ^79^ Table 5 offers an overview of the agents currently being investigated for MASLD/MASH.

**TABLE 5:** Advanced‐phase clinical trials investigating agents for the treatment of MASH and liver fibrosis.

Drug	Mode of action	Study	Phase
Aldafermin	FGF19 agonism	NCT02443116 NCT04210245 ALPINE 2/3	2B 2B 2B
Efruxifermin	FGF21 agonism	BALANCED HARMONY SYMMETRY SYNCHRONY	2 2 2 3
Pegozafermin	FGF21 agonism	ENLIVEN ENLIGHTEN	2 3
Pemvidutide	GLP1/glucagon dual agonist	NCT05006885	2
Cotadutide	GLP1/glucagon dual agonist	PROXYMO‐ADV	2
Survodutide	GL‐1/glucagon dual agonist	NCT04771273 NCT06309992	2 3
Retradutide	Triple agonist of GLP1/glucagon/gastric inhibitor peptide	NCT04881760	2
Lanifibrador	Pan‐PPAR agonism	NATIVE	3 (recruiting)
Firsocostat	Acetyl‐CoA Carboxylase inhibition	NCT02876796 NCT02856555	2 2
Clesacostat	Acetyl‐CoA Carboxylase inhibition	NCT03248882	2

*Note*: This Table describes different agents being currently investigated as treatment options for MASLD/MASH and liver fibrosis with their study‐names and the phase of the trials.

Abbreviations: Acetyl‐CoA, Acetyl coenzyme A; FGF, Fibroblast Growth Factor; GLP1, Glucagon‐like peptide 1; PPAR, Peroxisome proliferator‐activated receptor.

### 3.5 Effect of treatment on liver fibrosis

Ten trials evaluated the effect of antidiabetic treatment on liver fibrosis. Four evaluated the effect with TZD derivates.^27^, ^35^, ^36^, ^37^ Three reported on the effect of GLP‐1 agonists,^20^, ^68^, ^69^ two studies included SGLT2 inhibitors^51^, ^55^ and one DPP4 inhibitors.^44^

In the single arm study of Eguchi et al., 60% of the patients showed improvement of fibrosis. In the studies by Newsome and Armstrong there was improvement in fibrosis in the GLP1‐receptor agonist arm, but the results were not significant compared to the placebo group.^20^, ^68^

In the study of Bril et al. a significant reduction in fibrosis score was seen in the pioglitazone treatment arm compared to placebo.^37^ The three other trials which included an arm with TZD reported significant improvement of the NAFLD score, but no change in fibrosis or a non‐significant reduction of fibrosis.^27^, ^35^, ^36^

The study of Lai et al., which evaluated the effect of empagliflozin (SGLT2‐inhibitor) in a single arm trial showed a fibrosis improvement in 75% of the patients. The degree of change or improvement was not reported.^55^ The trial of Takahashi et al. found significantly more fibrosis improvement in the ipragliflozin group compared with the group with lifestyle modifications in combination with other antidiabetic drugs.^51^ In the study of Yilmaz et al., on the effects of sitagliptin no significant reduction in fibrosis was reported.^44^

## 4 DISCUSSION

This systematic literature review was conducted in accordance with PRISMA guidelines and provides insight in the effect of T2DM treatment on the severity of MASLD, MASH and MASH‐fibrosis. GLP‐1 receptor agonists, PPAR agonists and SGLT2 inhibitors may all be candidates for pharmacological treatment in patients with MASLD/MASH and T2DM.

We observed much heterogeneity in primary outcomes in the different trial designs. Outcomes were therefore inhomogeneous. Visceral adipose tissue, subcutaneous adipose tissue, CAP, FIB4, MRI‐PDFF and NALFD MRI scores were all used to evaluate the effect on MASLD. Ten trials evaluated the treatment effects with a histological reference, which is the golden standard for MASH.^80^ However, H‐MRS technique is the second best and can be considered as an acceptable alternative for liver biopsy. Part of the trials were pilot studies, including a small number of patients. The quality was therefore average. Due to small study populations or lack of power not all effect sizes could be calculated correctly. More recent studies used a more adequate design and included a placebo arm.^20^, ^35^, ^36^, ^37^, ^45^, ^50^, ^68^

Overall, the use of GLP‐1 receptor agonists, TZD derivates and SGLT2 inhibitors showed the strongest MASH improvements evaluated with liver biopsy or imaging techniques (CT and MRS). For the other medication groups, data was not clear. There are no placebo‐controlled trials evaluating the effect of insulin on MASLD/MASH, although some trials included insulin in the treatment‐arm. Insulin did not have a significant effect on MASLD/MASH in these studies, but different endpoints were used. Metformin, SU and DDP4‐inhibitors did not show consistent results.

To our knowledge this is one of the first systematic reviews to assess existing pharmacological T2DM drugs options for MASH/MASLD patients.

This systematic review has some limitations. A narrative design was chosen to deal with heterogeneity of the data. Due to differences in trial design, single arm trials and the lack of use of golden standard together with small sample sizes, effect sizes are small and variable. Due to this heterogeneity in studies, we deliberately chose to refrain from meta‐analysing the data quantitatively, since this might only generate spurious results. Even with the use of multiple medical search databases there were only few trials that used histological material or H‐MRS.

Another limitation of our study is that most research was done in males. MASLD is more prevalent in males, as androgens seem to promote pro‐inflammatory and cirrhotic pathways and estrogens appear to be protective.^81^ Although MASLD is more prevalent in males, MASH is more common in older women, and women are more likely to develop advanced liver disease.^82^, ^83^ The highest MASLD prevalence is in Latin America followed by the Middle East and North Africa.^84^ These differences can be partly explained by prevalence of metabolic comorbidities, genetic differences (like for example polymorphisms of the PNPLA3 gene), diet and socioeconomic factors.^83^ There seems to be no significant difference in prevalence of fibrosis in patients with MASLD according to ethnicity, but the available data are limited in this respect,^85^ since most studies have been done in white populations. Future research should take gender and ethnicity into account when investigating newer agents, to develop an optimal treatment strategy for each individual patient.

Various drugs used in the treatment of T2DM could be beneficial in patients with MASLD or MASH. While GLP1‐receptor agonists, SGLT2‐inhibitors and TZD‐derivatives may be most effective in this respect, the use of the latter may be hampered by potential cardiovascular safety profile issues, namely heart failure. In addition, both GLP1‐based therapy and SGLT2 inhibitors may have a more pronounced effect on the pathophysiological mechanisms involved in MASLD/MASH like the positive effects on body weight and the anti‐inflammatory effects. In addition, TZD‐derivatives increase insulin sensitivity through PPAR‐γ activity. GLP1‐receptor agonists may also have indirect effects on the liver, through reduced hepatopetal FFA‐flux from adipose tissue.^86^, ^87^, ^88^ Both, GLP1‐receptor agonists and SGLT2‐inhibitors have shown to decrease mortality and have a positive effect on preservation of kidney function.^89^ Whether part of these effects is mediated by the beneficial effects on the liver, is not yet clear.

To conclude, available evidence suggests improvements in liver enzymes and hepatic steatosis and fibrosis with GLP‐1 receptor agonists, SGLT‐2 inhibitors and TZD derivates. Therefore, these treatment options should be considered when dealing with MASLD/MASH patients, especially in the presence of T2DM. In the near future it is likely that newer agents like dual GLP‐1/GIP or even triple GLP‐1/GIP/Glucagon agonists will play a prominent role in treatment of patients with T2DM and MASLD/MASH. Given the presence of common drivers and shared pathophysiological mechanisms and since most patients with T2DM usually show a gradual increase in body weight, MASLD/MASH is a significant problem in patients with T2DM. Choosing medication with beneficial effects on both T2DM and MASLD will be of great relevance for these patients. Large placebo‐controlled clinical trials including a sufficient number of patients with an adequate follow up period are necessary to provide solid evidence for such dual efficacy.

## CONFLICT OF INTEREST

Authors declare no conflicts of interests.

## Supporting information


Data S1.

## Data Availability

The data underlying this article will be shared on reasonable request by the corresponding author.

## References

[1] European Association for the Study of the L, European Association for the Study of D, European Association for the Study of O . EASL‐EASD‐EASO clinical practice guidelines for the management of non‐alcoholic fatty liver disease. J Hepatol. 2016;64(6):1388‐1402. 27062661 10.1016/j.jhep.2015.11.004

[2] Younossi ZM , Koenig AB , Abdelatif D , Fazel Y , Henry L , Wymer M . Global epidemiology of nonalcoholic fatty liver disease‐meta‐analytic assessment of prevalence, incidence, and outcomes. Hepatology. 2016;64(1):73‐84. doi:10.1002/hep.28431 26707365

[3] Younossi ZM , Golabi P , de Avila L , et al. The global epidemiology of NAFLD and NASH in patients with type 2 diabetes: a systematic review and meta‐analysis. J Hepatol. 2019;71(4):793‐801. 31279902 10.1016/j.jhep.2019.06.021

[4] Samuel VT , Shulman GI . Nonalcoholic fatty liver disease, insulin resistance, and ceramides. N Engl J Med. 2019;381(19):1866‐1869. doi:10.1056/NEJMcibr1910023 31693811

[5] Buzzetti E , Linden A , Best LM , et al. Lifestyle modifications for nonalcohol‐related fatty liver disease: a network meta‐analysis. Cochrane Database Syst Rev. 2021;6(6):CD013156 doi:CD013156.pub2 [pii]. 34114650 10.1002/14651858.CD013156.pub2 PMC8193812

[6] Mantovani A , Csermely A , Petracca G , et al. Non‐alcoholic fatty liver disease and risk of fatal and non‐fatal cardiovascular events: an updated systematic review and meta‐analysis. Lancet Gastroenterol Hepatol. 2021;6(11):903‐913. 34555346 10.1016/S2468-1253(21)00308-3

[7] Ichikawa K , Miyoshi T , Osawa K , et al. Incremental prognostic value of non‐alcoholic fatty liver disease over coronary computed tomography angiography findings in patients with suspected coronary artery disease. Eur J Prev Cardiol. 2022;28(18):2059‐2066. doi:6323690 [pii]. 34279027 10.1093/eurjpc/zwab120

[8] Henry L , Paik J , Younossi ZM . Review article: the epidemiologic burden of non‐alcoholic fatty liver disease across the world. Aliment Pharmacol Ther. 2022;56(6):942‐956. doi:10.1111/apt.17158 35880713

[9] Eslam M, Sanyal AJ, George J, International Consensus P . MAFLD: a consensus‐driven proposed nomenclature for metabolic associated fatty liver disease. Gastroenterology. 2020;158(7):1999‐2014. 32044314 10.1053/j.gastro.2019.11.312

[10] Ziamanesh F , Mohammadi M , Ebrahimpour S , Tabatabaei‐Malazy O , Mosallanejad A , Larijani B . Unraveling the link between insulin resistance and non‐alcoholic fatty liver disease (or metabolic dysfunction‐associated steatotic liver disease): a narrative review. J Diabetes Metab Disord. 2023;22(2):1083‐1094. 37975107 10.1007/s40200-023-01293-3 PMC10638269

[11] Donnelly KL , Smith CI , Schwarzenberg SJ , Jessurun J , Boldt MD , Parks EJ . Sources of fatty acids stored in liver and secreted via lipoproteins in patients with nonalcoholic fatty liver disease. J Clin Invest. 2005;115(5):1343‐1351. 15864352 10.1172/JCI23621 PMC1087172

[12] Bhat N , Narayanan A , Fathzadeh M , et al. Dyrk1b promotes hepatic lipogenesis by bypassing canonical insulin signaling and directly activating mTORC2 in mice. J Clin Invest. 2022;132(3):e153724. doi:10.1172/JCI153724 34855620 PMC8803348

[13] Pierantonelli I , Svegliati‐Baroni G . Nonalcoholic fatty liver disease: basic Pathogenetic mechanisms in the progression from NAFLD to NASH. Transplantation. 2019;103(1):e1‐e13. doi:10.1097/TP.0000000000002480 30300287

[14] Rojano‐Toimil A , Rivera‐Esteban J , Manzano‐Nunez R , et al. When sugar reaches the liver: phenotypes of patients with diabetes and NAFLD. J Clin Med. 2022;11(12):3286. doi:10.3390/jcm11123286 35743358 PMC9225139

[15] Holmer M , Lindqvist C , Petersson S , et al. Treatment of NAFLD with intermittent calorie restriction or low‐carb high‐fat diet–a randomised controlled trial. JHEP Rep. 2021;3(3):100256. 33898960 10.1016/j.jhepr.2021.100256 PMC8059083

[16] Noto D , Petta S , Giammanco A , et al. Lifestyle versus ezetimibe plus lifestyle in patients with biopsy‐proven non‐alcoholic steatohepatitis (LISTEN): a double‐blind randomised placebo‐controlled trial. Nutr Metab Cardiovasc Dis. 2022;32(5):1288‐1291. 35256232 10.1016/j.numecd.2022.01.024

[17] Palmer AJ , Roze S , Valentine WJ , et al. Deleterious effects of increased body weight associated with intensive insulin therapy for type 1 diabetes: increased blood pressure and worsened lipid profile partially negate improvements in life expectancy. Curr Med Res Opin. 2004;20(Suppl 1):S67‐S73. 15324518 10.1185/030079904X2033

[18] Higgins JP , Altman DG , Gotzsche PC , et al. The Cochrane Collaboration's tool for assessing risk of bias in randomised trials. BMJ. 2011;343:d5928. 22008217 10.1136/bmj.d5928 PMC3196245

[19] Sterne JAC , Hernán MA , Reeves BC , et al. ROBINS‐I: a tool for assessing risk of bias in non‐randomised studies of interventions. BMJ. 2016;355:i4919. doi:10.1136/bmj.i4919 27733354 PMC5062054

[20] Newsome PN , Buchholtz K , Cusi K , et al. A placebo‐controlled trial of subcutaneous Semaglutide in nonalcoholic Steatohepatitis. N Engl J Med. 2020;384(12):1113‐1124. doi:10.1056/NEJMoa2028395 33185364

[21] Kuchay MS , Krishan S , Mishra SK , et al. Effect of dulaglutide on liver fat in patients with type 2 diabetes and NAFLD: randomised controlled trial (D‐LIFT trial). Diabetologia. 2020;63(11):2434‐2445. 32865597 10.1007/s00125-020-05265-7

[22] Kuchay MS , Krishan S , Mishra SK , et al. Effect of Empagliflozin on liver fat in patients with type 2 diabetes and nonalcoholic fatty liver disease: a randomized controlled trial (E‐LIFT trial). Diabetes Care. 2018;41(8):1801‐1808. 29895557 10.2337/dc18-0165

[23] Yoneda M , Honda Y , Ogawa Y , et al. Comparing the effects of tofogliflozin and pioglitazone in non‐alcoholic fatty liver disease patients with type 2 diabetes mellitus (ToPiND study): a randomized prospective open‐label controlled trial. BMJ Open Diabetes Res Care. 2021;9(1):e001990. doi:10.1136/bmjdrc-2020-001990 PMC7888333 33593749

[24] Yoneda M , Kobayashi T , Honda Y , et al. Combination of tofogliflozin and pioglitazone for NAFLD: extension to the ToPiND randomized controlled trial. Hepatol Commun. 2022;6(9):2273‐2285. 35578445 10.1002/hep4.1993 PMC9426404

[25] Feng W , Gao C , Bi Y , et al. Randomized trial comparing the effects of gliclazide, liraglutide, and metformin on diabetes with non‐alcoholic fatty liver disease. J Diabetes. 2017;9(8):800‐809. doi:10.1111/1753-0407.12555 28332301

[26] Fan H , Pan Q , Xu Y , Yang X . Exenatide improves type 2 diabetes concomitant with non‐alcoholic fatty liver disease. Arq Bras Endocrinol Metabol. 2013;57(9):702‐708. 24402015 10.1590/s0004-27302013000900005

[27] Omer Z , Cetinkalp S , Akyildiz M , et al. Efficacy of insulin‐sensitizing agents in nonalcoholic fatty liver disease. Eur J Gastroenterol Hepatol. 2010;22(1):18‐23. doi:10.1097/MEG.0b013e32832e2baf 19667999

[28] Komorizono Y , Hosoyamada K , Imamura N , et al. Metformin dose increase versus added linagliptin in non‐alcoholic fatty liver disease and type 2 diabetes: an analysis of the J‐LINK study. Diabetes Obes Metab. 2021;23(3):832‐837. doi:10.1111/dom.14263 33236464

[29] Wang X , Zhao B , Sun H , You H , Qu S . Effects of sitagliptin on intrahepatic lipid content in patients with non‐alcoholic fatty liver disease. Front Endocrinol (Lausanne). 2022;13:866189. doi:10.3389/fendo.2022.866189 36072931 PMC9441565

[30] Jiang X , Shi T , Han D , Chen J . Exenatide and metformin improve serum indices and intestinal Flora in patients with type 2 diabetes mellitus and non‐alcoholic fatty liver disease. J Pak Med Assoc. 2024;74(1):138‐140. 38219182 10.47391/JPMA.8295

[31] Shi M , Zhang H , Wang W , et al. Effect of dapagliflozin on liver and pancreatic fat in patients with type 2 diabetes and non‐alcoholic fatty liver disease. J Diabetes Complicat. 2023;37(10):108610. 10.1016/j.jdiacomp.2023.108610 37722211

[32] Hooshmand Gharabagh L , Shargh A , Mohammad Hosseini Azar MR , Esmaeili A . Comparison between the effect of Empagliflozin and pioglitazone added to metformin in patients with type 2 diabetes and nonalcoholic fatty liver disease. Clin Res Hepatol Gastroenterol. 2024;48(3):102279 doi:S2210‐7401(23)00204‐8 [pii]. 38159676 10.1016/j.clinre.2023.102279

[33] Kinoshita T , Shimoda M , Nakashima K , et al. Comparison of the effects of three kinds of glucose‐lowering drugs on non‐alcoholic fatty liver disease in patients with type 2 diabetes: a randomized, open‐label, three‐arm, active control study. J Diabetes Investig. 2020;11(6):1612‐1622. 10.1111/jdi.13279 PMC7610105 32329963

[34] Lee YH , Kim JH , Kim SR , et al. Lobeglitazone, a novel thiazolidinedione, improves non‐alcoholic fatty liver disease in type 2 diabetes: its efficacy and predictive factors related to responsiveness. J Korean Med Sci. 2017;32(1):60‐69. 27914133 10.3346/jkms.2017.32.1.60 PMC5143300

[35] Cusi K , Orsak B , Bril F , et al. Long‐term pioglitazone treatment for patients with nonalcoholic Steatohepatitis and prediabetes or type 2 diabetes mellitus: a randomized trial. Ann Intern Med. 2016;165(5):305‐315. 27322798 10.7326/M15-1774

[36] Belfort R , Harrison SA , Brown K , et al. A placebo‐controlled trial of pioglitazone in subjects with nonalcoholic steatohepatitis. N Engl J Med. 2006;355(22):2297‐2307. 17135584 10.1056/NEJMoa060326

[37] Bril F , Kalavalapalli S , Clark VC , et al. Response to pioglitazone in patients with nonalcoholic Steatohepatitis with vs without type 2 diabetes. Clin Gastroenterol Hepatol. Apr 2018;16(4):558‐566. doi:S1542‐3565(17)31424‐6 [pii]. doi:10.1016/j.cgh.2017.12.001 29223443

[38] Ito D , Shimizu S , Inoue K , et al. Comparison of Ipragliflozin and pioglitazone effects on nonalcoholic fatty liver disease in patients with type 2 diabetes: a randomized, 24‐week, open‐label, active‐controlled trial. Diabetes Care. 2017;40(10):1364‐1372. 28751548 10.2337/dc17-0518

[39] Cho KY , Nakamura A , Omori K , et al. Favorable effect of sodium‐glucose cotransporter 2 inhibitor, dapagliflozin, on non‐alcoholic fatty liver disease compared with pioglitazone. J Diabetes Investig. 2021;12(7):1272‐1277. 10.1111/jdi.13457 PMC8264405 33131199

[40] Han E , Huh JH , Lee EY , et al. Efficacy and safety of evogliptin in patients with type 2 diabetes and non‐alcoholic fatty liver disease: a multicentre, double‐blind, randomized, comparative trial. Diabetes Obes Metab. 2022;24(4):752‐756. doi:10.1111/dom.14623 34918436

[41] Zhang LY , Qu XN , Sun ZY , Zhang Y . Effect of liraglutide therapy on serum fetuin a in patients with type 2 diabetes and non‐alcoholic fatty liver disease. Clin Res Hepatol Gastroenterol. 2020;44(5):674‐680. 32113823 10.1016/j.clinre.2020.01.007

[42] Morteza Aghajanpoor MG , Mosaed R . The efficacy of Empagliflozin in combination with pioglitazone on the improvement of fatty liver disease in patients with type 2 diabetes. Annals of military and health sciences. Research. 2024;22(2):1‐5. doi:10.5812/amh-144009.

[43] Khaliq A , Badshah H , Shah Y , et al. The effect of ertugliflozin in patients with nonalcoholic fatty liver disease associated with type 2 diabetes mellitus: a randomized controlled trial. Medicine (Baltimore). 2024;103(45):e40356. 39533572 10.1097/MD.0000000000040356 PMC11556963

[44] Yilmaz Y , Yonal O , Deyneli O , Celikel CA , Kalayci C , Duman DG . Effects of sitagliptin in diabetic patients with nonalcoholic steatohepatitis. Acta Gastroenterol Belg. 2012;75(2):240‐244. 22870790

[45] Cui J , Philo L , Nguyen P , et al. Sitagliptin vs. placebo for non‐alcoholic fatty liver disease: a randomized controlled trial. J Hepatol. 2016;65(2):369‐376. 27151177 10.1016/j.jhep.2016.04.021 PMC5081213

[46] Kim J , Han K , Kim B , et al. Sodium‐glucose cotransporter 2 inhibitors for non‐alcoholic fatty liver disease in patients with type 2 diabetes mellitus: a nationwide propensity‐score matched cohort study. Diabetes Res Clin Pract. 2022;194:110187. 36442545 10.1016/j.diabres.2022.110187

[47] Hiruma S , Shigiyama F , Kumashiro N . Empagliflozin versus sitagliptin for ameliorating intrahepatic lipid content and tissue‐specific insulin sensitivity in patients with early‐stage type 2 diabetes with non‐alcoholic fatty liver disease: a prospective randomized study. Diabetes Obes Metab. 2023;25(6):1576‐1588. doi:10.1111/dom.15006 36749298

[48] Yan J , Yao B , Kuang H , et al. Liraglutide, Sitagliptin, and insulin glargine added to metformin: the effect on body weight and intrahepatic lipid in patients with type 2 diabetes mellitus and nonalcoholic fatty liver disease. Hepatology. 2019;69(6):2414‐2426. 30341767 10.1002/hep.30320 PMC6594101

[49] Sumida Y , Murotani K , Saito M , et al. Effect of luseogliflozin on hepatic fat content in type 2 diabetes patients with non‐alcoholic fatty liver disease: a prospective, single‐arm trial (LEAD trial). Hepatol Res. 2019;49(1):64‐71. doi:10.1111/hepr.13236 30051943

[50] Shimizu M , Suzuki K , Kato K , et al. Evaluation of the effects of dapagliflozin, a sodium‐glucose co‐transporter‐2 inhibitor, on hepatic steatosis and fibrosis using transient elastography in patients with type 2 diabetes and non‐alcoholic fatty liver disease. Diabetes Obes Metab. 2019;21(2):285‐292. doi:10.1111/dom.13520 30178600

[51] Takahashi H , Kessoku T , Kawanaka M , et al. Ipragliflozin improves the hepatic outcomes of patients with diabetes with NAFLD. Hepatol Commun. 2022;6(1):120‐132. 34558835 10.1002/hep4.1696 PMC8710792

[52] Bellanti F , Lo Buglio A , Dobrakowski M , et al. Impact of sodium glucose cotransporter‐2 inhibitors on liver steatosis/fibrosis/inflammation and redox balance in non‐alcoholic fatty liver disease. World J Gastroenterol. 2022;28(26):3243‐3257. doi:10.3748/wjg.v28.i26.3243 36051336 PMC9331534

[53] Eriksson JW , Lundkvist P , Jansson PA , et al. Effects of dapagliflozin and n‐3 carboxylic acids on non‐alcoholic fatty liver disease in people with type 2 diabetes: a double‐blind randomised placebo‐controlled study. Diabetologia. 2018;61(9):1923‐1934. 29971527 10.1007/s00125-018-4675-2 PMC6096619

[54] Elhini SH , Wahsh EA , Elberry AA , et al. The impact of an SGLT2 inhibitor versus Ursodeoxycholic acid on liver steatosis in diabetic patients. Pharmaceuticals (Basel). 2022;15(12):1516. doi:10.3390/ph15121516 36558967 PMC9786599

[55] Lai LL , Vethakkan SR , Nik Mustapha NR , Mahadeva S , Chan WK . Empagliflozin for the treatment of nonalcoholic Steatohepatitis in patients with type 2 diabetes mellitus. Dig Dis Sci. 2020;65(2):623‐631. 30684076 10.1007/s10620-019-5477-1

[56] Inoue M , Hayashi A , Taguchi T , et al. Effects of canagliflozin on body composition and hepatic fat content in type 2 diabetes patients with non‐alcoholic fatty liver disease. J Diabetes Investig. 2019;10(4):1004‐1011. 10.1111/jdi.12980 PMC6626966 30461221

[57] Tobita H , Sato S , Miyake T , Ishihara S , Kinoshita Y . Effects of Dapagliflozin on body composition and liver tests in patients with nonalcoholic Steatohepatitis associated with type 2 diabetes mellitus: a prospective, open‐label, uncontrolled study. Curr Ther Res Clin Exp. 2017;87:13‐19. 28912902 10.1016/j.curtheres.2017.07.002 PMC5587885

[58] Kahl S , Gancheva S , Strassburger K , et al. Empagliflozin effectively lowers liver fat content in well‐controlled type 2 diabetes: a randomized, double‐blind, phase 4. Placebo‐Controlled Trial Diabetes Care Feb. 2020;43(2):298‐305. doi:dc19‐0641 [pii]. doi:10.2337/dc19-0641 31540903

[59] Hussain MTS , Babar ZM . Therapeutic outcome of dapagliflozin on various parameters in non‐alcoholic fatty liver disease (NAFLD) patients. Int J Diabetes Dev Ctries. 2022;42:290‐296.

[60] Borisov AN , Kutz A , Christ ER , Heim MH , Ebrahimi F . Canagliflozin and metabolic associated fatty liver disease in patients with diabetes mellitus: new insights from CANVAS. J Clin Endocrinol Metab. 2023;108(11):2940‐2949. 37149821 10.1210/clinem/dgad249 PMC10584001

[61] Phrueksotsai S , Pinyopornpanish K , Euathrongchit J , et al. The effects of dapagliflozin on hepatic and visceral fat in type 2 diabetes patients with non‐alcoholic fatty liver disease. J Gastroenterol Hepatol. 2021;36(10):2952‐2959. doi:10.1111/jgh.15580 34129252

[62] Cusi K , Bril F , Barb D , et al. Effect of canagliflozin treatment on hepatic triglyceride content and glucose metabolism in patients with type 2 diabetes. Diabetes Obes Metab. 2019;21(4):812‐821. doi:10.1111/dom.13584 30447037

[63] Guo W , Tian W , Lin L , Xu X . Liraglutide or insulin glargine treatments improves hepatic fat in obese patients with type 2 diabetes and nonalcoholic fatty liver disease in twenty‐six weeks: a randomized placebo‐controlled trial. Diabetes Res Clin Pract. 2020;170:108487. 33035599 10.1016/j.diabres.2020.108487

[64] Liu L , Yan H , Xia M , et al. Efficacy of exenatide and insulin glargine on nonalcoholic fatty liver disease in patients with type 2 diabetes. Diabetes Metab Res Rev. 2020;36(5):e3292. doi:10.1002/dmrr.3292 31955491

[65] Shao N , Kuang HY , Hao M , Gao XY , Lin WJ , Zou W . Benefits of exenatide on obesity and non‐alcoholic fatty liver disease with elevated liver enzymes in patients with type 2 diabetes. Diabetes Metab Res Rev. 2014;30(6):521‐529. doi:10.1002/dmrr.2561 24823873

[66] Gastaldelli A , Cusi K , Fernandez Lando L , Bray R , Brouwers B , Rodriguez A . Effect of tirzepatide versus insulin degludec on liver fat content and abdominal adipose tissue in people with type 2 diabetes (SURPASS‐3 MRI): a substudy of the randomised, open‐label, parallel‐group, phase 3 SURPASS‐3 trial. Lancet Diabetes Endocrinol. 2022;10(6):393‐406. 35468325 10.1016/S2213-8587(22)00070-5

[67] Galicia‐Garcia U , Benito‐Vicente A , Jebari S , et al. Pathophysiology of type 2 diabetes mellitus. Int J Mol Sci. 2020;21(17):6275. doi:10.3390/ijms21176275 32872570 PMC7503727

[68] Armstrong MJ , Gaunt P , Aithal GP , et al. Liraglutide safety and efficacy in patients with non‐alcoholic steatohepatitis (LEAN): a multicentre, double‐blind, randomised, placebo‐controlled phase 2 study. Lancet. 2016;387(10019):679‐690. doi:S0140‐6736(15)00803‐X [pii]. 26608256 10.1016/S0140-6736(15)00803-X

[69] Eguchi Y , Kitajima Y , Hyogo H , et al. Pilot study of liraglutide effects in non‐alcoholic steatohepatitis and non‐alcoholic fatty liver disease with glucose intolerance in Japanese patients (LEAN‐J). Hepatol Res. 2015;45(3):269‐278. doi:10.1111/hepr.12351 24796231

[70] Parker VER , Robertson D , Erazo‐Tapia E , et al. Cotadutide promotes glycogenolysis in people with overweight or obesity diagnosed with type 2 diabetes. Nat Metab. 2023;5(12):2086‐2093. 38066113 10.1038/s42255-023-00938-0 PMC10730390

[71] Volpe S , Lisco G , Fanelli M , et al. Once‐weekly subcutaneous Semaglutide improves fatty liver disease in patients with type 2 diabetes: a 52‐week prospective real‐life study. Nutrients. 2022;14(21):4673. doi:10.3390/nu14214673. 36364937 PMC9657108

[72] Arai T , Atsukawa M , Tsubota A , et al. Beneficial effect of oral semaglutide for type 2 diabetes mellitus in patients with metabolic dysfunction‐associated steatotic liver disease: a prospective, multicentre, observational study. Diabetes Obes Metab. 2024;26(11):4958‐4965. doi:10.1111/dom.15898 39223865

[73] Hu Y , Sun C , Chen Y , Liu YD , Fan JG . Pipeline of new drug treatment for non‐alcoholic fatty liver disease/metabolic dysfunction‐associated Steatotic liver disease. J Clin Transl Hepatol. 2024;12(9):802‐814. [pii] 10.14218/JCTH.2024.00123. 39280073 10.14218/JCTH.2024.00123 PMC11393841

[74] Patel RH , Parikh C , Upadhyay H , et al. Resmetirom in the Management of Metabolic Dysfunction‐Associated Steatohepatitis (MASH): a comprehensive review of current evidence and therapeutic potential. Cureus. 2024;16(11):e74772. doi:10.7759/cureus.74772 39735033 PMC11682840

[75] Harrison SA , Rolph T , Knott M , Dubourg J . FGF21 agonists: an emerging therapeutic for metabolic dysfunction‐associated steatohepatitis and beyond. J Hepatol. 2024;81(3):562‐576. 38710230 10.1016/j.jhep.2024.04.034

[76] Marey MM , Belal M , Awad AA , et al. Efficacy and safety of aldafermin in non‐alcoholic steatohepatitis: a systematic review and meta‐analysis of randomized controlled trials. Clin Res Hepatol Gastroenterol. 2024;48(6):102357. 38688423 10.1016/j.clinre.2024.102357

[77] Ciardullo S , Muraca E , Vergani M , Invernizzi P , Perseghin G . Advancements in pharmacological treatment of NAFLD/MASLD: a focus on metabolic and liver‐targeted interventions. Gastroenterol Rep (Oxf). 2024;12:goae029. doi:10.1093/gastro/goae029 38681750 PMC11052658

[78] Puengel T , Tacke F . Pharmacotherapeutic options for metabolic dysfunction‐associated steatotic liver disease: where are we today? Expert Opin Pharmacother. 2024;25(9):1249‐1263. doi:10.1080/14656566.2024.2374463 38954663

[79] Sanyal AJ , Kaplan LM , Frias JP , et al. Triple hormone receptor agonist retatrutide for metabolic dysfunction‐associated steatotic liver disease: a randomized phase 2a trial. Nat Med. 2024;30(7):2037‐2048. 38858523 10.1038/s41591-024-03018-2 PMC11271400

[80] Ando Y , Jou JH . Nonalcoholic fatty liver disease and recent guideline updates. Clin Liver Dis. 2021;17(1):23‐28. 10.1002/cld.1045 PMC7849298 33552482

[81] Lee C , Kim J , Jung Y . Potential therapeutic application of Estrogen in gender disparity of nonalcoholic fatty liver disease/nonalcoholic Steatohepatitis. Cells. 2019;8:cells‐08‐01259. 10.3390/cells8101259 PMC6835656 31619023

[82] Talens M , Tumas N , Lazarus JV , Benach J , Pericas JM . What do we know about inequalities in NAFLD distribution and outcomes? A scoping review. J Clin Med. 2021;10(21):5019. doi:10.3390/jcm10215019 34768539 PMC8584385

[83] Miller KC , Geyer B , Alexopoulos AS , Moylan CA , Pagidipati N . Disparities in metabolic dysfunction‐associated Steatotic liver disease prevalence, diagnosis, treatment, and outcomes: a narrative review. Dig Dis Sci. 2024;70(1):154‐167. doi:10.1007/s10620-024-08722-0 39560808 PMC13318092

[84] Younossi ZM , Golabi P , Paik JM , Henry A , Van Dongen C , Henry L . The global epidemiology of nonalcoholic fatty liver disease (NAFLD) and nonalcoholic steatohepatitis (NASH): a systematic review. Hepatology. 2023;77(4):1335‐1347. 36626630 10.1097/HEP.0000000000000004 PMC10026948

[85] Rich NE , Oji S , Mufti AR , et al. Racial and ethnic disparities in nonalcoholic fatty liver disease prevalence, severity, and outcomes in the United States: a systematic review and meta‐analysis. Clin Gastroenterol Hepatol. 2018;16(2):198‐210. 28970148 10.1016/j.cgh.2017.09.041 PMC5794571

[86] Thomas MK , Nikooienejad A , Bray R , et al. Dual GIP and GLP‐1 receptor agonist Tirzepatide improves Beta‐cell function and insulin sensitivity in type 2 diabetes. J Clin Endocrinol Metab. 2021;106(2):388‐396. 33236115 10.1210/clinem/dgaa863 PMC7823251

[87] Jiang Y , Wang Z , Ma B , et al. GLP‐1 improves adipocyte insulin sensitivity following induction of endoplasmic reticulum stress. Original research. Front Pharmacol. 2018;9:1168. doi:10.3389/fphar.2018.01168 30459598 PMC6232689

[88] Smith U , Kahn BB . Adipose tissue regulates insulin sensitivity: role of adipogenesis, de novo lipogenesis and novel lipids. J Intern Med. 2016;280(5):465‐475. 27699898 10.1111/joim.12540 PMC5218584

[89] Palmer SC , Tendal B , Mustafa RA , et al. Sodium‐glucose cotransporter protein‐2 (SGLT‐2) inhibitors and glucagon‐like peptide‐1 (GLP‐1) receptor agonists for type 2 diabetes: systematic review and network meta‐analysis of randomised controlled trials. BMJ. 2021;372:m4573. 33441402 10.1136/bmj.m4573 PMC7804890

