# Supplementary material for: Pharmacological treatment options for metabolic dysfunction‐associated steatotic liver disease in patients with type 2 diabetes mellitus: A systematic review
Source: Eur J Clin Invest. 2025 Feb 12;55(4):e70003. doi: 10.1111/eci.70003 (PMC11891831; doi:10.1111/eci.70003)
Supplement: Supplementary file 1 — Data S1. [file ECI-55-e70003-s001.docx]

**Supplementary Files**

**Supplementary S1:** Literature search: strategy for PubMed (NCBI)

("Non-alcoholic Fatty Liver Disease"[Mesh] OR Non-alcoholic Fatty Liver*[tiab] OR nonalcoholic fatty liver*[tiab] OR NAFLD [tiab] OR nonalcoholic steatohepatit*[tiab] OR non-alcoholic steatohepatit*[tiab] OR NASH [tiab] OR Metabolic Dysfunction-Associated steatotic liver disease [tiab] OR Metabolic Dysfunction-Associated steatohepatitis [tiab] OR MASLD [tiab] OR MASL [tiab] OR MASH [tiab]) AND ("Diabetes Mellitus"[Mesh] OR diabet*[tiab]) AND ("Drug Therapy"[Mesh] OR "Hypoglycemic Agents"[Mesh] OR drug therap*[tiab] OR drug treatment*[tiab] OR pharmacotherap*[tiab] OR pharmacological*[tiab]) AND English[lang]

**Supplementary figure 1** Risk of bias assessment according to Cochrane assessment




Legend: 41 studies were assigned as an RCT according for the 5 risk of bias domains; D1 bias arising of the
randomization process, D2 bias due to deviations from intended interventions, D3 bias due to missing outcome data, D4 bias due to measurements of outcome, D5 bias in selection of reported results. The color represent the judgement in final or per domain: red (high concerns), yellow (some concern), green (low concern).

**Supplementary figure 2** Risk of bias assessment according to Cochrane assessment


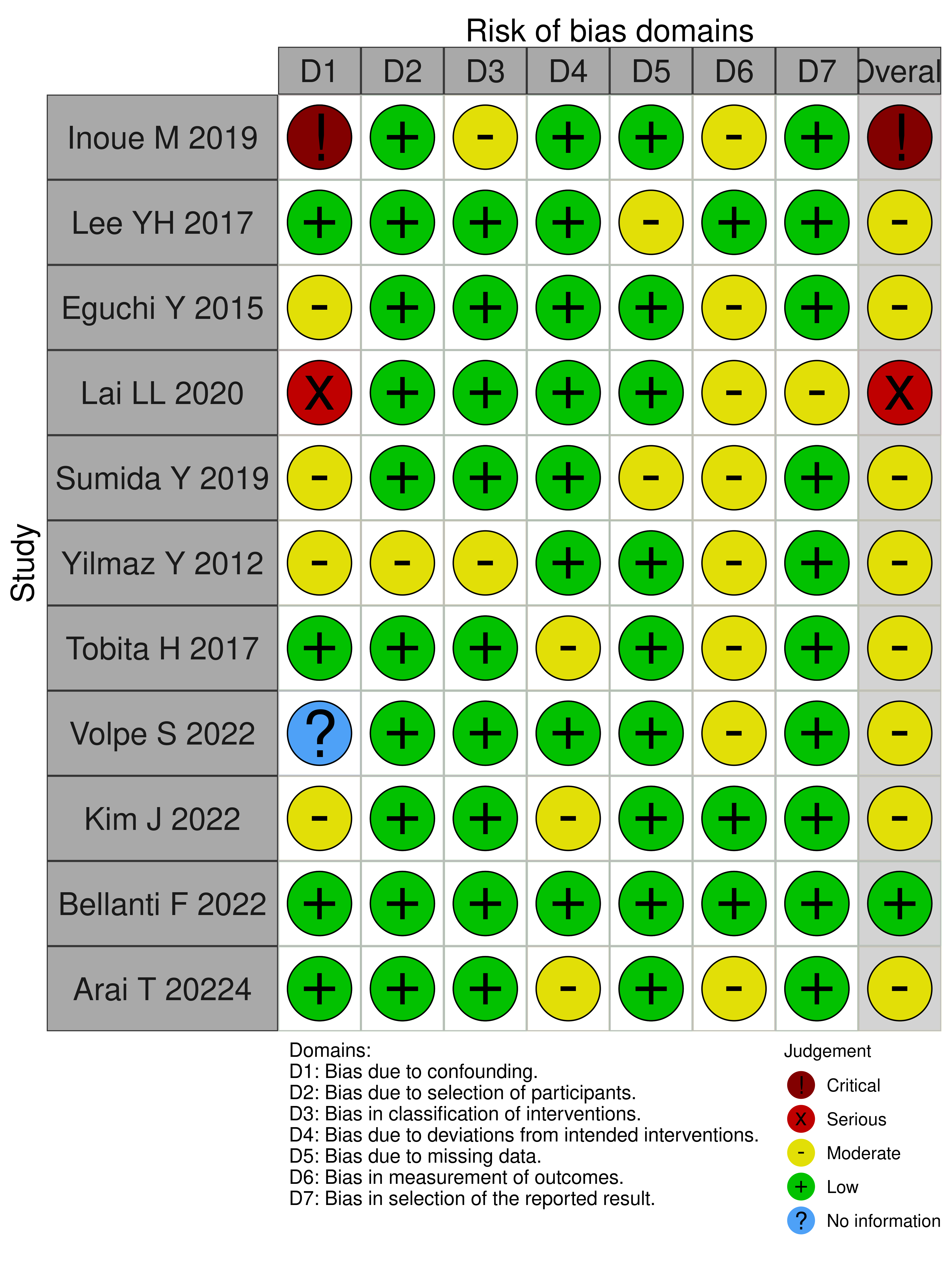


Legend: 11 studies accessed according the ROBINS-1 Cochrane tools for 7 domains: Bias due to confounding, Bias in selection of participants into the study, Bias in classification of interventions, Bias due to deviations from intended interventions, Bias due to missing data, Bias in measurement of outcomes, Bias in selection of the reported result. The color represent the judgement in final or per domain: red (high concerns), orange (high concerns), yellow (some concern), green (low concern).
